# Mechanical Properties, Physical Properties and VOC Emissions of Three-Layer Particleboards with Recycled Automotive Plastics in the Core Layer

**DOI:** 10.3390/polym17111438

**Published:** 2025-05-22

**Authors:** Anna Darabošová, Tatiana Bubeníková, Iveta Čabalová, Miroslav Badida, Çağrı Olgun, Önder Tor, Mustafa Öncel

**Affiliations:** 1 Department of Chemistry and Chemical Technologies, Faculty of Wood Sciences and Technology, Technical University in Zvolen, 960 01 Zvolen, Slovakia; xdarabosova@is.tuzvo.sk (A.D.); bubenikova@tuzvo.sk (T.B.); 2 Department of Business Management and Economics, Institute of Industrial Engineering, Management and Applied Mathematics, Faculty of Mechanical Engineering, Technical University of Košice, 042 00 Košice, Slovakia; miroslav.badida@tuke.sk; 3 Department of Forest Industry Engineering, Faculty of Forestry, Kastamonu University, 37210 Kastamonu, Turkey; colgun@kastamonu.edu.tr (Ç.O.); ondertor@kastamonu.edu.tr (Ö.T.); moncel@kastamonu.edu.tr (M.Ö.)

**Keywords:** wood–plastic composites, mechanical properties, physical properties, VOC emissions

## Abstract

The growing volume of plastic waste from end-of-life vehicles presents environmental concerns, driving efforts to integrate recycled plastics. This study investigates the possibility of using recycled plastic from automotive parts (painted and unpainted bumpers, fuel tanks) as a 10% filler in the core layer of three-layer particleboards (P) and evaluates its impact on physical properties (water absorption—WA and thickness swelling—TS), mechanical properties (internal bonding strength—IB, modulus of rupture—MOR, modulus of elasticity—MOE and screw driving torque—SDT) and volatile organic compounds—VOC emissions. The boards were produced using conventional hot-pressing technology and analyzed according to applicable standards. Based on the results, the density of the reference (P) was 0.72 g·cm^−3^, while wood–plastic composites ranged from 0.70 g·cm^−3^ to 0.72 g·cm^−3^. After 24 h, WA reached 40% for reference (P) and from 36.9% (for (P) containing unpainted bumpers) to 41.9% (for (P) containing fuel tanks). TS reached 18% for (P) and from 16.8% (for (P) containing unpainted bumpers and fuel tanks) to 18.1% (for (P) containing painted bumpers). Plastic is a hydrophobic material and it is assumed that by increasing the proportion of plastic filler in the particleboards, the WA and TS of prepared boards will decrease. From the point of view of mechanical properties, values for (P) containing plastic filler were slightly lower compared to reference (P). The lowest value of IB (0.39 MPa) were reached for (P) containing painted bumpers. Plastic surface treatment could interfere with adhesion between the plastic and adhesive, weakening the bond in the core layer. For this reason, is preferable to use unpainted fillers, which provide better adhesive properties and higher structural integrity. VOC emissions from wood components consisted primarily of monoterpenes such as α-pinene, 3-carene and limonene. Adding 10% plastic to the particleboard did not increase overall VOC emissions. On the other hand, combining wood and plastic particles resulted in a reduction in overall VOC emissions. The findings confirm that recycled automotive plastics can be effectively incorporated into particleboards, maintaining standard performance while reducing reliance on virgin wood materials, making them a viable and sustainable alternative for furniture and interior applications.

## 1. Introduction

Particleboards are extensively used in the production of furniture and various wooden products, including decorative profiles, cabinets, kitchen countertops, and interior construction applications. These engineered materials are manufactured from lignocellulosic raw materials such as wood particles, chips, and sawdust, which are bonded into boards using thermosetting adhesives under heat and pressure. Among these adhesives, urea-formaldehyde (UF) resin is the most commonly employed due to its rapid curing time and excellent bonding properties. A typical example is three-layer particleboard, comprising surface layers made of fine wood particles and a core of coarser chips [1,2].

Compared to solid wood, particleboards offer distinct advantages, including reduced defects and the ability to produce boards in customizable sizes and densities. Significant advancements in particleboard manufacturing have focused on producing low-density boards with superior mechanical and physical properties. These efforts aim to optimize wood utilization, reducing consumption while enhancing board quality, thereby supporting the sustainable management of forest resources and protecting global ecosystems [3].

In recent decades, wood–polymer composites (WPCs) have emerged as a highly promising class of hybrid materials with applications in construction (e.g., decking and cladding), infrastructure, and transportation. The performance of WPCs is influenced by wood content, processing methods, and additives. Timber industry by-products, such as pine and spruce, are commonly utilized as raw materials [4,5,6]. Advances in product design, manufacturing technologies, and alternative material applications have driven the steady growth of the WPC market [6,7].

WPCs are innovative composite materials that synergistically combine wood fibers and polymeric plastics, offering exceptional strength, durability, and sustainability [8]. These materials find widespread use in construction, automotive industry, and furniture manufacturing, providing longer service life and reduced maintenance compared to solid wood [7]. Incorporating wood flour into a polypropylene matrix reduces production costs while enhancing mechanical properties [9]. The production process involves blending wood fillers with molten polymers, pelletizing the mixture, and shaping the material through extrusion, injection molding, or compression molding [10]. Research underscores the ability of WPCs to combine environmental sustainability with outstanding technical performance, making them a viable alternative to conventional building materials [11].

The composition of WPCs typically includes a polymer matrix—commonly polypropylene, polyethylene, or polyvinylchloride—wood fibers, and additives such as lubricants, pigments, and flame retardants [12]. Manufacturing occurs at processing temperatures below the thermal degradation threshold of wood, ensuring the integrity of wood particles [13]. These materials hold significant potential for recycling and repurposing waste, reducing environmental impacts [11].

While WPCs exhibit benefits such as water resistance, durability, and insect resistance, they show limitations in bending strength, sunlight tolerance, and heat resistance. These drawbacks can be mitigated using chemical additives, although this increases costs and reduces ecological acceptability [14]. The mechanical properties of WPCs depend on factors such as fiber size, binder agents, and polymer type. Smaller fibers improve impact strength, and mixing different fiber sizes enhances overall mechanical performance [15]. For example, Khamedi et al. [16] found that composites containing 40% sawdust, 56% polyethylene, and 3% maleic anhydride achieved optimal tensile strength, suggesting that moderate wood reinforcement improves properties compared to higher wood content.

The use of recycled polymers in WPCs offers environmental and economic benefits. Studies demonstrate that composites made with recycled high-density polyethylene (HDPE) have comparable mechanical properties to those made with virgin HDPE [17]. Adding coupling agents and fibers such as bagasse improves mechanical performance to levels similar to virgin materials [18]. Adhikary et al. [19] and Kazemi-Najafi et al. [20] showed that recycled HDPE and PP composites can perform as well as or better than virgin plastics. Additionally, Jayaraman and Halliwell [21] developed strong, formable composites from waste plastics and flax fibers.

However, WPCs can release volatile organic compounds (VOCs), potentially affecting indoor air quality and health. VOCs such as formaldehyde, terpenes, and aldehydes are emitted from adhesives, additives, and the materials themselves [22,23]. The emission of volatile organic compounds (VOCs) from wood-based panels is a significant concern due to potential health risks associated with indoor air quality. Comprehensive reviews have highlighted various methods for evaluating these emissions and strategies for their mitigation [24]. Although high manufacturing temperatures can reduce emissions, VOCs may still be released post-production, particularly in new buildings or rooms with substantial WPC use [25]. While research indicates that typical VOC emissions from WPCs do not pose significant health risks under normal usage (Felix et al. [26]), further studies are needed to assess long-term exposure impacts [27].

The environmental sustainability of WPCs is complemented by their enhanced properties, such as improved moisture resistance, reduced water absorption, and dimensional stability. Incorporating automotive plastic waste into composites can further enhance physical properties, such as minimizing thickness swelling [28]. Additionally, WPCs exhibit better resistance to weather conditions, making them ideal for outdoor use [29]. Combining advancements in particleboard and WPC technologies allows the development of hybrid composites that integrate waste materials from timber and polymer industries, creating sustainable engineered materials with broader applications. A similar approach has been demonstrated by Mancel et al. [30], who successfully incorporated waste rubber materials into three-layer particleboards, evaluating their physical and mechanical performance.

The density of particleboard significantly affects its quality [3]. Xu et al. [31] studied low-density binderless particleboards made from kenaf core and observed excellent mechanical properties despite their low density. Boards with a density of 0.20 g·cm^−3^ exhibited a modulus of rupture of 1.1 MPa, modulus of elasticity of 0.3 GPa, and good dimensional stability.

This work focuses on recycling plastics from end-of-life cars. These are materials that are very difficult to recycle due to their complicated structure. Plastic fuel tanks are most commonly manufactured from HDPE, which is characterized by its low weight, high strength, and chemical resistance to substances such as gasoline and diesel. Additionally, this material offers excellent impact resistance and easy moldability, allowing manufacturers to create complex designs that maximize fuel capacity and ensure optimal integration into vehicles [32,33,34]. Besides HDPE, polypropylene (PP) is also widely used in the automotive industry, particularly for bumper production, due to its favorable cost, lightweight nature, and good mechanical properties [35,36]. Despite these advantages, plastic waste represents a significant environmental burden due to pollution, soil degradation, and the release of microplastics, which negatively impact human and animal health [37,38,39].

Recycling plastic waste from the automotive industry, which generates 8–9 million tons of end-of-life vehicle waste annually in Europe alone, offers an innovative solution to mitigate these negative impacts [40]. Incorporating recycled automotive plastics, such as used HDPE fuel tanks or PP bumpers, into three-layer particleboard composites reduces ecological burdens while simultaneously optimizing the properties of the resulting materials.

This study focused on developing and evaluating novel three-layer wood–plastic composites made from spruce wood particles and automotive plastic waste incorporated into the core layer. The research assessed the impact of these waste plastics on physical properties (e.g., thickness swelling, water absorption, density), mechanical properties (e.g., internal bonding strength, bending strength, seating torque, and stripping torque), and environmental aspects (e.g., VOC emissions). By examining these factors, the study demonstrated the feasibility of using automotive plastic waste for production of wood–plastic composites, balancing material quality with environmental sustainability.

## 2. Materials and Methods

### 2.1. Material

Three-layer wood-based composite materials were produced by incorporating automotive waste polymers into particleboards (P). The particleboards were manufactured using wood particles obtained from spruce logs processed at Kronospan s.r.o. in Zvolen, Slovakia. The wood particles used in the core layer ranged in size from 0.25 mm to 4.0 mm, while the particles used in the surface layer were finer, between 0.25 mm and 1.0 mm. All wood particles were dried to achieve a moisture content of 5% before being used in the production process.

The automotive waste plastics were provided by ALUEX s.r.o. in Zvolen, Slovakia. These plastics included polypropylene (PP) bumpers, both painted and unpainted, and high-density polyethylene (HDPE) fuel tanks sourced from decommissioned vehicles. These waste plastics were cut into smaller pieces, cleaned, and subsequently processed into granules using the plastic shredding equipment at the Technical University of Zvolen. Granulation was carried out using a plastic shredder DP 11–240/350 (Profing, Piešťany, Slovakia) and a dust extractor ABS 1080 (Holzmann Maschinen, Haslach, Austria). Granules (size from 1 mm to 4 mm) were sieved using an analytical sieve AS 200 digit cA (Retsch, Haan, Germany) to ensure uniform particle size.

For the binder, urea-formaldehyde (UF) resin-Kronores CB 1100 F (Diakol Strážske, s.r.o., Strážske, Slovakia) was used, which contains 67.1% solids and has a viscosity of 460 mPa.s and a gel time of 55 s, with a pH of 8.6. To facilitate the curing process, ammonium nitrate (NH_4_NO_3_, concentration of 47%) was employed as a hardener, along with a 30% paraffin emulsion to improve the water resistance and durability of the final product.

**Figure 1:**
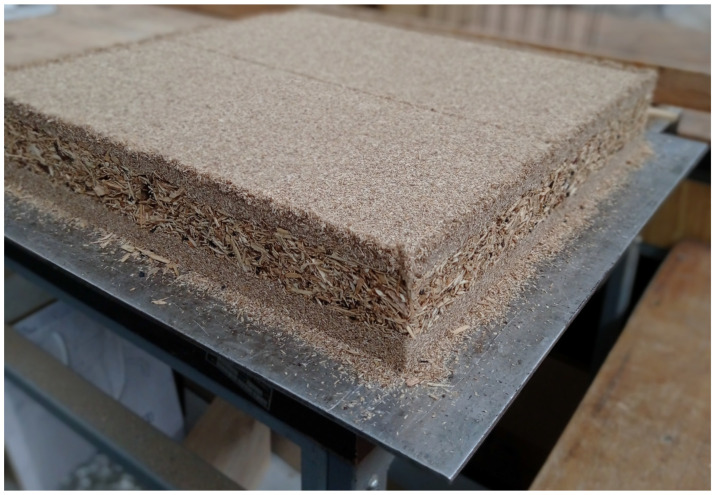
Prepared particleboard mat before pressing.

The manufacturing process followed standard particleboard production technology. The first step involved preparing particle mats, which were initially cold-pressed at a pressure of 1 MPa (Figure 1). After this, the mats were hot-pressed using a CBJ 100–11 laboratory press (TOS, Rakovník, Czech Republic). The surface layers of the boards were formed by mixing wood particles with the adhesive binder, while the core layer was composed of a combination of wood particles, crushed plastic granules, and adhesive. These materials were thoroughly mixed to ensure a uniform distribution of the plastics within the core.

The manufacturing of the boards involved specific proportions of materials: 396 g of the surface layer mixture was placed into a mold, followed by 1110 g of the core layer mixture. The final step involved placing another 396 g of surface layer material on top. Exact quantities of all components used in the production of particleboards containing plastic additives are presented in Table 1. This mold assembly was then cold pre-pressed for 2 min, followed by hot pressing at a temperature of 230 °C using a constant hydraulic pressure of 5 MPa for a total pressing time of 5 min. The pressing duration was slightly extended due to the inclusion of waste plastics granulate in the core layer. The full production methodology followed the procedure outlined in utility model no. 10249 [41].

The boards containing waste plastic from automobiles, incorporated at a 10% proportion relative to the core layer mass, were produced in dimensions of 360 × 360 × 18 mm.

**Figure 2:**
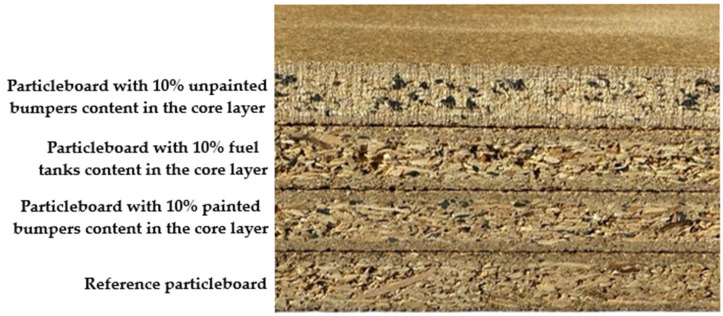
Samples of reference particleboard and particleboards containing plastic fillers.

The resulting three-layer particleboards were produced with three different types of plastic fillers, including painted bumpers (PB10), unpainted bumpers (UB10), and fuel tanks (FT10) (Figure 2). Each filler type was incorporated at a content level of 10%. In addition, a three-layer particleboard without any plastic filler (P) was also produced, which serves as the reference board for comparison. The designations of all samples are provided in Table 2.

Prior to the production of these three-layer particleboards, extensive research was conducted to determine the optimal percentage of plastic material for this type of composite. Initial studies involved the production of single-layer boards with varying amounts of recycled plastic incorporated into the material. The aim was to assess the effect of different plastic content on the board’s mechanical and physical properties. Several plastic content percentages were tested, including 10%, 15%, and 20%. The results indicated that 10% plastic content yielded the best overall performance in terms of strength, durability, and other key properties.

Since the 10% plastic content showed the best performance, this formulation was chosen for further development. Instead of producing a single-layer board, a three-layer particleboard was manufactured, where the core layer essentially consisted of the single-layer formulation with 10% recycled plastic. This approach allowed the optimal plastic content to be combined with the structural advantages of the three-layer design, thereby improving the overall properties of the composite material.

The preliminary results regarding the 10%, 15%, and 20% plastic content in the single-layer boards are available in the published articles. These studies provide detailed insights into the performance and properties of the boards at different plastic content levels [42,43,44].

### 2.2. Methods

#### 2.2.1. Density

The density of each sample was determined using the gravimetric method in accordance with the ASTM D2395-17 (2017) standard [45]. Measurements were conducted on six samples for each particleboard using 50 mm × 50 mm × 18 mm (width × length × thickness) specimens from each board. The dimensions (thickness, width, and length) were accurately measured with a digital caliper to calculate the volume, while the mass was recorded. The density (ρ) was calculated according to the following equation:

$$\rho =\frac{m}{V}$$

where *m* is the sample mass and *V* is the calculated volume. This method ensured reliable and precise density measurements, with the final density value obtained as the average of the individual sample densities.

#### 2.2.2. Water Absorption and Thickness Swelling

The D1037-12 (2020) standard [46] was used to determine water absorption and thickness swelling after 2 and 24 h of immersion. Measurements were conducted on eight samples for each particleboard. The samples were submerged at least 25 mm below the water surface in a water bath (Nüve BM 402, NÜVE SANAYİ MALZEMELERİ İMALAT VE TİCARET A.Ş., Ankara, Turkey) filled with clean water maintained at 20 °C, ensuring they remained separated from each other and the container walls. The water was replaced after each test.

After 2 h and 24 h, the samples were removed, and their thickness and weight were measured. Thickness swelling was recorded as the change in thickness, while water absorption was determined based on the change in weight, using the initial thickness and weight as reference values. The water absorption and thickness swelling were calculated according to the following equation:

$$G_{t}=\frac{t2-t1}{t1}\times 100\;(\%)$$

where *t*2 is the value of the sample after immersion in grams for WA and in millimeters for TS, and *t*1 is the value of the sample before immersion in grams for WA and in millimeters for TS.

#### 2.2.3. Mechanical Properties

The mechanical properties of the samples were assessed by determining their internal bonding strength (IB) and bending strength, modulus of rupture (MOR), and modulus of elasticity (MOE). Testing was conducted using a Shimadzu AG-IC 20/50 KN STD Universal Testing Machine (Star Teknik Elektronik Cihazlar Sanayi, Ticaret, Turkey). The test device is calibrated periodically and the last calibration before the analysis of the test samples was made one year before measurement.

Internal bonding strength was determined following ASTM-D1037-12 (2020) standard [46] on eight samples using 50 mm × 50 mm × 18 mm (width × length × thickness) specimens from each board. Specimens were bonded to blocks using an appropriate adhesive and subjected to an increasing load until failure occurred perpendicular to the plane of the material. The maximum load was reached within 60 s, and IB was calculated based on the maximum force applied to the specimen surface, using the average cross-sectional area and thickness as input parameters. The internal bonding strength was calculated according to the following equation:

$$f_{t}=\frac{Fmax}{a\cdot b}\;(MPa)$$

where *Fmax* is load acting on the sample at failure in newtons, *a* is length of the sample in millimeters, and *b* is width of the sample in millimeters.

**Figure 3:**
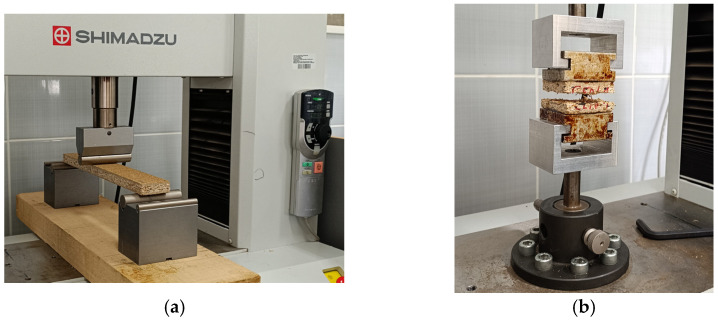
Testing of mechanical properties of particleboards: (**a**) three-point bending test of particleboard using a Shimadzu universal testing machine; (**b**) tensile strength testing of particleboard samples.

Bending strength was measured according to ASTM-D1037-12 (2020) standard [46], where six samples were placed on two supports and subjected to a central load of a 50 mm × 460 mm × 18 mm (width × length × thickness) specimen of each board with a grip of 50 mm and a span of 360 mm. The bending strength was calculated as the ratio of the bending moment at the breaking load to the section modulus, with input parameters including the average width at the loading point and thickness. The methodology of mechanical properties is shown in Figure 3.

The modulus of rupture (MOR) and modulus of elasticity (MOE) was calculated according to the following equation:

$$f_{m}=\frac{3\cdot Fmax\cdot L}{2\cdot b\cdot t^{2}}\;(MPa)$$

where f_m_ is Modulus of Rupture, *Fmax* is breaking load in newtons, *L* is distance between the centers of the supports in millimeters, *b* is width of the sample in millimeters, and *t* is thickness of the sample in millimeters.

$$E_{m}=\frac{L^{3}\cdot (F2-F1)}{4\cdot b\cdot t^{3}\cdot (a2-a1)}\;(MPa)$$

where E_m_ is Modulus of Elasticity, *L* is distance between the centers of the supports in millimeters, *F*2 − *F*1 is load increase in newtons at elastic deformation area, *b* is width of the sample in millimeters, *t* is thickness of the sample in millimeters, and *a*2 − *a*1 is deflection increase at half the length of the sample in millimeters at elastic deformation area.

Additionally, the screw driving torque (SDT) was measured, specifically the seating torque (SeT) and stripping torque (StT). The test involved inserting a screw into the sample and monitoring the torque during rotation without resistance. Measurements were conducted on eight samples for each particleboard.

Statistical analysis was performed using multifactor analysis of variance (ANOVA) and DUNCAN tests to examine differences in water absorption, thickness swelling, internal bonding, modulus of rupture, modulus of elasticity, seating torque, and stripping torque values. The analyses were conducted using the SPSS 23 pocket program (IBM, Armonk, NY, USA).

#### 2.2.4. Volatile Organic Compounds

**Figure 4:**
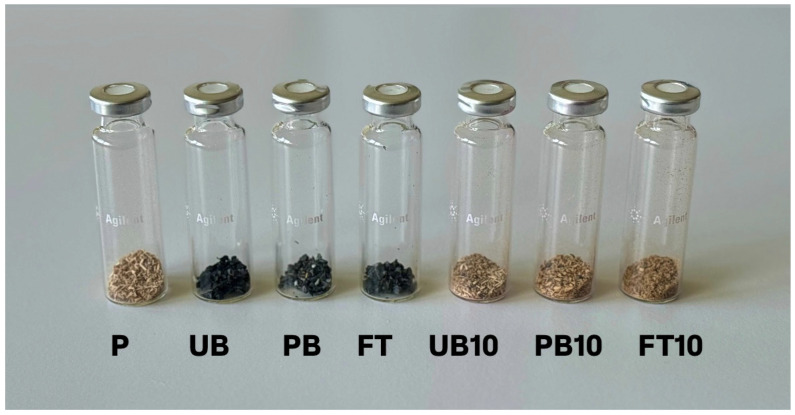
Samples of wood particles, reference particleboard, plastics, and composite boards with 10% plastic content, prepared for VOC analysis via GC-MS. Each vial contains precisely 0.5 g of the sample and is hermetically sealed with a silicone septum to ensure controlled headspace conditions: P—reference particleboard; UB—unpainted bumpers; PB—painted bumpers; FT—fuel tanks; UB10—particleboard with 10% unpainted bumpers; PB10—particleboard with 10% painted bumpers; FT10—particleboard with 10% fuel tanks.

The emission of volatile organic compounds (VOCs) from newly prepared wood–plastic composite materials was evaluated using Headspace Gas Chromatography-Mass Spectrometry (HS-GC-MS). Samples weighing 0.5 g were placed in 20 mL headspace vials, which were sealed with aluminum caps and PTFE/silicone septa (Figure 4). The vials were then thermally conditioned at four different temperatures using a headspace apparatus. During thermal exposure, the volatile compounds emitted from the samples were separated by gas chromatography and identified using mass spectrometry.

The analysis was performed in four distinct tests with varying conditions. In each test, helium was used as the carrier gas at a pressure of 7.5 psi. The oven temperature for Test 1 was set to 35 °C, with the loop at 45 °C and the transfer line at 55 °C. In Test 2, the oven temperature was raised to 50 °C, the loop to 60 °C, and the transfer line to 70 °C. For Test 3, the oven temperature was set to 100 °C, the loop to 110 °C, and the transfer line to 120 °C. Finally, in Test 4, the oven temperature was 150 °C, the loop was set to 160 °C, and the transfer line was at 170 °C. In each case, the sample was equilibrated for 60 min before analysis.

Gas chromatography was conducted with a headspace injector set to 180 °C, with a split ratio of 20:1. The column used was HP-5MS (30 m × 0.250 mm × 0.25 μm), and the carrier gas was helium, flowing at a constant rate of 1.0 mL/min. The temperature program for the column was set from 40 °C to 270 °C, with the transfer line maintained at 280 °C.

Mass spectrometry conditions: the scan range was 45 amu to 500 amu, the ion source temperature was 230 °C, and the quadrupole temperature was 150 °C. VOCs were identified by comparing the obtained spectra with the NIST17 mass spectral library.

In this study, VOC emissions were analyzed from various materials relevant to composite production, three types of automotive plastics (fuel tanks, painted bumpers, and unpainted bumpers), a reference particleboard and wood–plastic composites containing 10% of these plastics in the core layer. Each sample type was analyzed once under each test condition. For better clarity, the abbreviations of the materials used are explained in Table 3.

## 3. Results and Discussion

### 3.1. Physical Properties

#### 3.1.1. Density of Prepared Particleboards

The experiment was conducted to determine the density of the tested specimens using the gravimetric method. Table 4 provides average density values. The experimentally determined density of the tested wood–plastic composites ranged between 0.70 g·cm^−3^ and 0.72 g·cm^−3^, corresponding to typical values for standard particleboards. The average density of pure spruce wood is approximately 0.45 g·cm^−3^ [47], whereas the density of pressed particleboards typically increases by about 5–40% compared to the density of the wood used [48]. The density of the plastic components utilized is higher: high-density polyethylene (HDPE), used in fuel tanks, ranges between 0.954 g·cm^−3^–0.96 g·cm^−3^, while polypropylene (PP), commonly used for bumpers, has a density of approximately 0.90 g·cm^−3^ to 0.91 g·cm^−3^ [49]. Clearly, the inclusion of plastic components contributes to the increased overall density of the composites.

#### 3.1.2. Water Absorption and Thickness Swelling of Prepared Particleboards 

The results of the ANOVA and Duncan analyses for water absorption (WA) and thickness swelling (TS) after 2 and 24 h are presented in Table 5. Based on the values, it can be concluded that the average WA after 2 h ranged from 10.8% to 12.8%, the average WA after 24 h ranged from 36.9% to 41.9%, the average TS after 2 h ranged from 4.7% to 5.6%, and TS after 24 h ranged from 16.8% to 18.1%. ANOVA analysis showed that there was no statistically significant difference between the 2 and 24-h thickness swelling (TS) and water absorption (WA) groups. The absence of statistically significant changes in water absorption and thickness swelling values (2 h and 24 h) indicates that waste automobile parts can be easily integrated into the particleboard production line, maintaining uniform quality in dimensional stability.

In comparison with other studies, water absorption and thickness swelling in wood–plastic composites (WPCs) and modified particleboards have been reported to vary significantly based on composition. For instance, an increase in the recycled wood particle (RWP) content in WPCs led to a notable rise in water absorption and thickness swelling, with values for WPCs containing 60% RWPs being 2.7 times and 2.8 times higher than those with 20% RWPs after 24 h [50]. Similarly, the reduction of polymeric methylene diphenyl diisocyanate (PMDI) content from 4% to 2.8% resulted in a significant increase in short-term water absorption (WA) and thickness swelling (TS), indicating the crucial role of adhesive content in moisture resistance. However, the addition of high-density polyethylene (HDPE) has been shown to substantially improve water resistance, as it forms a hydrophobic barrier that prevents moisture penetration into the particleboard structure [51].

Compared to these studies, the results in this work suggest that the tested material maintains relatively stable dimensional properties over time, making it a viable alternative for sustainable particleboard production. The lack of significant changes between 2 h and 24 h WA and TS further confirms the material’s ability to resist moisture uptake, which is a key factor in ensuring long-term performance in practical applications.

#### 3.1.3. Mechanical Properties of Prepared Particleboards

**Figure 5:**
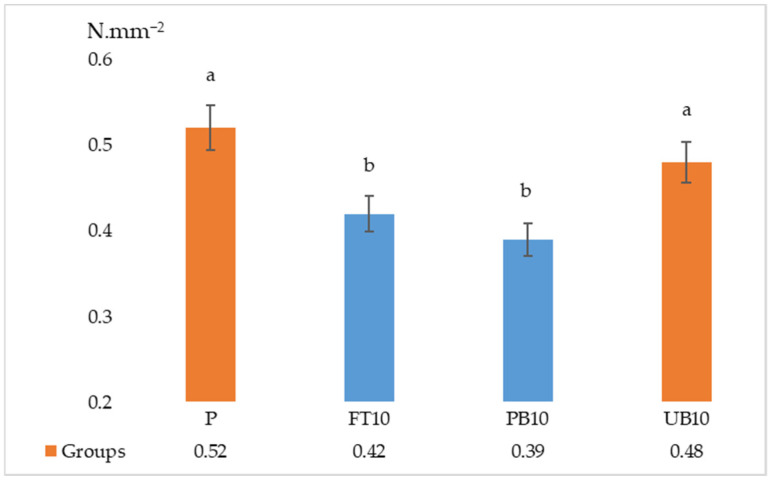
Results of internal bonding strength of particleboards containing plastic filler (P—reference particleboard; FT10—particleboard containing 10% waste fuel tanks; PB10—particleboard containing 10% waste painted bumpers; UB10—particleboard containing 10% waste unpainted bumpers). Note: Different letters indicate different homogeneity groups.

**Figure 6:**
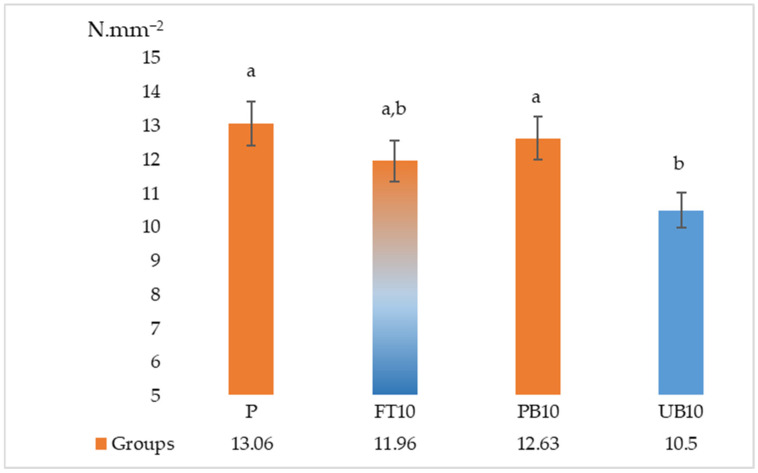
Results of modulus of rupture of particleboards containing plastic filler (P—reference particleboard; FT10—particleboard containing 10% waste fuel tanks; PB10—particleboard containing 10% waste painted bumpers; UB10—particleboard containing 10% waste unpainted bumpers). Note: Different letters indicate different homogeneity groups.

**Figure 7:**
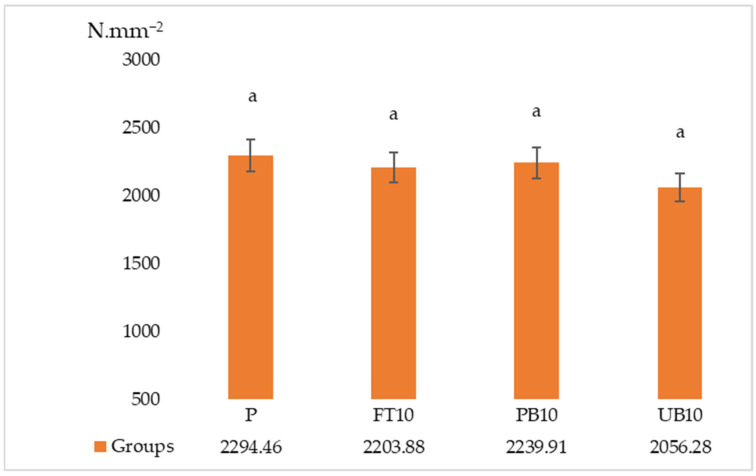
Results of modulus of elasticity of particleboards containing plastic filler (P—reference particleboard; FT10—particleboard containing 10% waste fuel tanks; PB10—particleboard containing 10% waste painted bumpers; UB10—particleboard containing 10% waste unpainted bumpers). Note: Different letters indicate different homogeneity groups.

**Figure 8:**
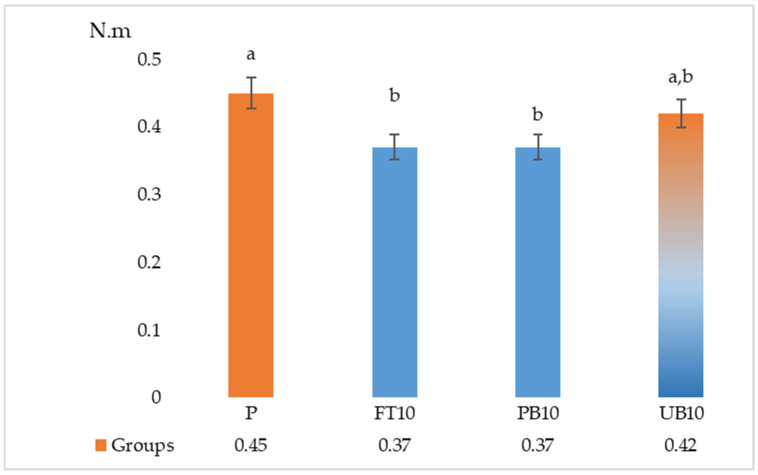
Results of seating torque of particleboards containing plastic filler (P—reference particleboard; FT10—particleboard containing 10% waste fuel tanks; PB10—particleboard containing 10% waste painted bumpers; UB10—particleboard containing 10% waste unpainted bumpers). Note: Different letters indicate different homogeneity groups.

**Figure 9:**
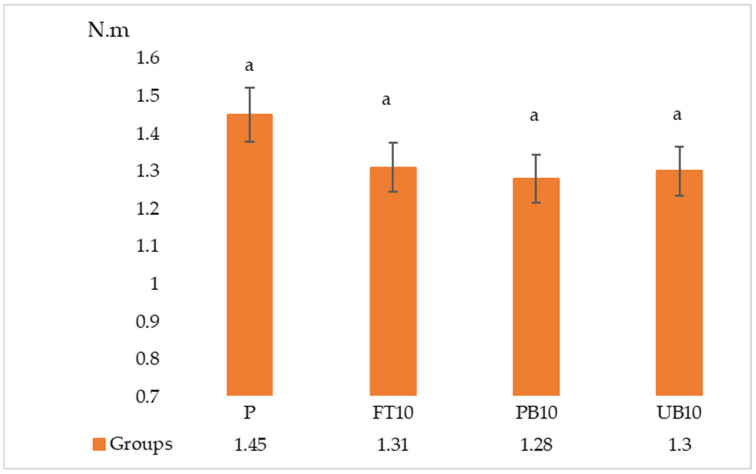
Results of stripping torque of particleboards containing plastic filler (P—reference particleboard; FT10—particleboard containing 10% waste fuel tanks; PB10—particleboard containing 10% waste painted bumpers; UB10—particleboard containing 10% waste unpainted bumpers). Note: Different letters indicate different homogeneity groups.

The results of the ANOVA and Duncan analyses for internal bonding (IB), modulus of rupture (MOR), and modulus of elasticity (MOE) are presented in Table 6. Graphical representations of these results are shown in Figure 5, Figure 6, Figure 7, Figure 8 and Figure 9. The results indicate that the average internal bond (IB) strength varied from 0.39 MPa to 0.52 MPa, the average modulus of rupture (MOR) ranged from 10.50 MPa to 13.06 MPa, the average modulus of elasticity (MOE) spanned from 2056.28 MPa to 2294.46 MPa, the average seating torque (SeT) fluctuated between 0.37 N·m and 0.45 N·m, and the average stripping torque (StT) ranged from 1.28 N·m to 1.45 N·m. ANOVA analysis showed a statistically significant difference in the MOR, IB strength, and SeT strength groups, but no statistically significant difference was observed in the MOE and StT strength groups.

Based on the results, it can be stated that the highest IB strength property appeared in the samples with no addition of waste polymers, and the lowest IB strength property appeared in samples with the addition of painted bumper waste.

When the data were analyzed, it was observed that there is no significant differences between the IB values of the reference group and unpainted bumper samples. However, there is a statistical difference between the particle boards with painted bumper added and the group with unpainted bumper added. It can be concluded that the paint waste negatively affects internal bonding strength.

The statistical analysis indicates no significant change in the MOE values between the reference sample and the groups with added waste automotive components. No significant difference was found between the reference group and the groups with fuel tank and painted bumper waste for MOR values. It suggests that waste automotive parts can be utilized in the production of standard quality particle board, considering MOE and MOR values.

When comparing these results with previous studies, it is evident that the mechanical properties of WPC and P strongly depend on composition and processing methods. Chaharmahali et al. [52] found that MOE reached its peak at 70% fiber content (2093 MPa), while it decreased to 1864 MPa at 60% and 1753 MPa at 80% fiber content. Similarly, MOR was highest at 70% fiber content (14.5 MPa) but dropped to 12.3 MPa at 80% fiber content, which was attributed to decreased compatibility between the wood and polymer phases. This trend aligns with the results of this study, where adding waste polymers did not lead to significant changes in mechanical performance.

A similar pattern was observed by Bütün et al. [53], who demonstrated that WPC made from MDF (middle density fiberboard) recycled via thermo-hydro-mechanical disintegration exhibited higher MOE (2750 MPa) and MOR (23.4 MPa) compared to mechanically recycled WPC (MOE = 2580 MPa, MOR = 20.8 MPa). Additionally, the addition of MAPP (maleic anhydride-modified polypropylene) improved flexural strength by up to 12% and impact resistance by 15%, emphasizing the role of coupling agents in optimizing WPC mechanical properties.

The study by Kamdem et al. [54] further confirmed that WPC containing HDPE and CCA-treated wood particles achieved the highest MOE (995.1 MPa) and MOR (18.83 MPa). In contrast, recycled particleboards exhibited lower mechanical performance, with MOE dropping to 498.5 MPa and MOR to 9.93 MPa, which is consistent with the findings of this study, where samples containing waste polymers and painted surfaces showed reduced internal bond strength (IB ranging from 0.39 MPa to 0.52 MPa).

These findings suggest that waste automotive parts can be successfully integrated into particleboard production without significantly compromising MOE and MOR. However, the decrease in internal bond strength (IB) may be linked to surface treatments or reduced adhesion between materials. Furthermore, the results confirm that the mechanical properties of particleboard and WPC can be optimized by selecting appropriate recycled materials and coupling agents, which could be crucial for the future development of these materials.

According to the test results, it can be stated that the highest SeT property appeared in samples with no addition of waste polymers, the lowest SeT property appeared in board samples with addition of waste unpainted bumper and fuel tank groups, the highest StT property appeared in samples with no addition of waste polymers, and the lowest StT property appeared in samples with addition of painted bumper.

When the data were analyzed, no significant differences were observed among the groups regarding StT properties; it is believed that the additives used in production did not significantly change the StT.

Compared to previous studies, Yu et al. [55] reported SeT values from 0.66 N·m to 1.94 N·m and StT values from 2.03 N·m to 6.51 N·m, while Tor et al. [56] found particleboard’s StT values ranging from 3.12 N·m to 6.05 N·m. The lower torque values in this study, especially in samples with waste polymers, may be due to differences in core density, particle size, and IB strength.

Previous research also shows that SeT and StT are higher when screws are inserted into particleboard faces rather than edges, and pilot holes reduce these values. While this study did not compare edge vs. face performance, the lower StT in painted samples suggests reduced adhesion and screw-holding capacity.

Overall, the addition of waste automotive materials affects torque performance, with painted surfaces negatively impacting StT. Further optimization of particle size and adhesion properties could improve screw retention in particleboard formulations containing recycled materials.

#### 3.1.4. Volatile Organic Compounds Emitted from Prepared Particleboards

Volatile organic compounds (VOCs) are released from both natural and synthetic materials, especially under elevated temperatures [57]. Understanding their emission behavior is crucial for assessing the environmental and health impacts of polymer-based composites.

To evaluate the influence of temperature on VOC release, samples were tested at four different temperatures: 35 °C, 50 °C, 100 °C, and 150 °C. The results are presented in graphical form, providing a comparative overview of emission trends across different material types and temperature conditions. The findings highlight the differences in emission profiles between wood-based and polymer-based components, as well as the effect of incorporating automotive plastics into composite structures.

**Figure 10:**
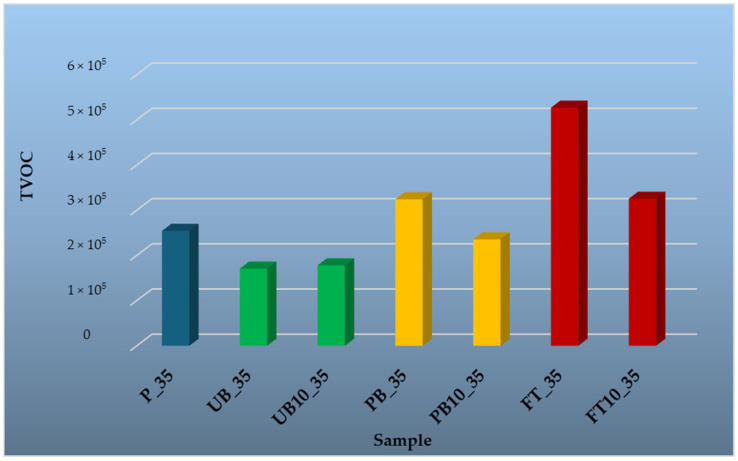
Comparison of total peak areas of volatile organic compounds (TVOC) emitted from waste automobile plastics, reference particleboard and particleboards containing plastic filler at a temperature of 35 °C.

Figure 10 presents the results of volatile organic compound (VOC) analysis for various materials at a temperature of 35 °C. At a temperature of 35 °C, compared to other samples, the lowest amount of VOCs was emitted from unpainted bumpers (UB_35) and from particleboard with 10% unpainted bumpers (UB10_35). In the sample of unpainted bumpers (UB_35), p-xylene was identified at 7.52% of the total TVOC. This is a significant chemical feedstock used in various industrial processes, including large-scale polymer synthesis. No other compounds were identified in this sample.

In the sample of painted bumpers (PB_35), several volatile compounds were identified. o-xylene (4.4%) is primarily used for the production of o-dicarboxylic acid anhydride, which finds applications in plasticizers, resins, and dyes [58]. Additionally, 1,2,3-trimethylbenzene (4.92%) was present, which forms via thermal decomposition of limonene, commonly found in plastics and rubber and can be of both natural and synthetic origin [59,60].

The highest TVOC content was recorded in the fuel tank sample (FT_35). Identified compounds included p-xylene (2.09%), 1,2,3-trimethylbenzene (5.66%), and 1,2,4-trimethylbenzene (4.95%), the latter of which is commonly found in fuels [61]. o-cymene (3.43%) was also detected, which occurs in trace amounts in monoterpene fractions of resins and in extracts and essential oils from various parts of coniferous trees [62].

Samples of the reference particleboard analyzed at 35 °C (P_35) showed the presence of only a few volatile organic compounds: α-pinene (11.04%), β-pinene (2.89%)—well-known volatile compounds of natural origin, primarily derived from the wood component of the material and recognized as key VOCs emitted from particleboard [63,64,65,66]—and 3-carene (1.60%), which is the second most abundant monoterpene in pine after α-pinene. Other common monoterpenes found in pine include β-pinene, limonene, and terpinene [67,68].

Compared to particleboards containing plastic waste from automobiles, the samples with unpainted bumpers (UB10_35), painted bumpers (PB10_35), and fuel tanks (FT10_35) contained only wood-derived terpenes. In the UB10_35 sample, α-pinene (9.50%) was identified. The PB10_35 sample contained α-pinene (11.13%) and 3-carene (5.25%). In the FT10_35 sample, α-pinene (21.37%) and β-pinene (5.77%) were detected.

Since no compounds typically associated with plastics—previously identified in the analysis of individual plastic components—were detected in the samples containing plastic waste, it is likely that these emissions are suppressed by the presence of wood. The volatile compounds from wood may mask or inhibit the release of VOCs from plastics, thus dominating the overall emission profile of the material. Only selected volatile organic compounds are discussed in the text; the full list of identified VOCs is available in Table S1 (Supplementary Materials).

**Figure 11:**
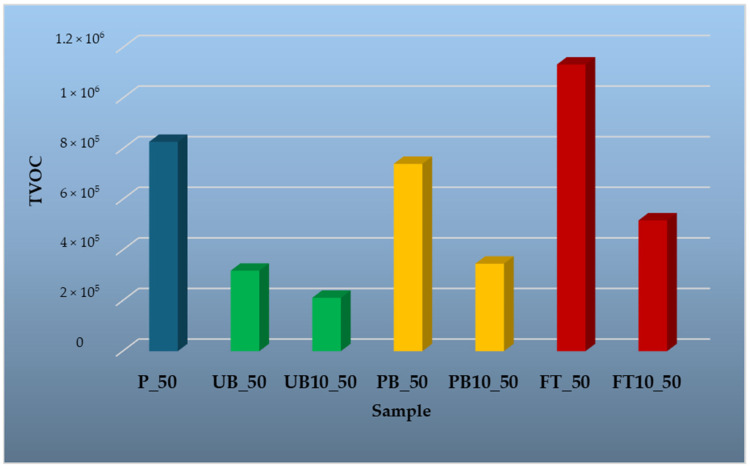
Comparison of total peak areas of volatile organic compounds (TVOC) emitted from waste automobile plastics, reference particleboard and particleboards containing plastic filler at a temperature of 50 °C.

Figure 11 presents the analysis of volatile organic compounds (VOCs) at an elevated temperature of 50 °C. As expected, the total VOC emissions increased compared to 35 °C in all tested materials. For samples P, PB and FT, the increase was more than twofold. In contrast, particleboard samples with 10% content of these plastics (PB10_50, UB10_50, FT10_50) did not show such a marked increase in VOCs. In the UB_50 plastic sample, only p-xylene (9.68%) and ethylbenzene (3.71%) were identified. Ethylbenzene, along with toluene—found in PB_50 and FT_50 samples—are residual volatile solvents resulting from polymerization [69]. In the PB_50 plastic sample, besides toluene (1.03%), p-xylene (3.81%) and o-xylene (1.27%) were identified, similar to the analysis at 35 °C. Further compounds detected included styrene (0.74%), a basic raw material for polymer production and an important chemical in the petrochemical industry [70], mesitylene (0.99%), α-methylstyrene (1.54%), a derivative of styrene [70], 1,2,3-trimethylbenzene (4.96%), 2,3,6-trimethyltetradecane (12.43%), 3,6-dimethyldecane (3.12%), 2,3,5,8-tetramethyldecane (4.03%), and 1-iodotridecane (4.09%). The plastic sample from fuel tanks (FT_50) again exhibited the highest total VOC content, similar to the results at 35 °C. Several of the compounds identified at the lower temperature were also detected at 50 °C, along with additional volatile organic compounds. Compounds that appeared only at 50 °C included toluene (0.51%), or naphthalene (1.46%)—a toxic air pollutant formed by biomass and petroleum combustion as well as industrial production [71], and methenamine (1.45%), which is used in the production of plastics and resins [72].

The reference particleboard sample at 50 °C contained α-pinene (53.12%), β-pinene (9.84%), and 3-carene (5.20%). These compounds were also present in particleboards with added plastic filler—PB10_50 contained α-pinene (29.78%), β-pinene (3.89%), and 3-carene (11.00%); in the UB10_50 sample, only α-pinene (13.97%) was identified, and in FT10_50, α-pinene (46.17%) and β-pinene (5.41%) were found.

These results confirm that wood-based composites with recycled polymer additives do not exhibit significant VOC emissions even at 50 °C. Despite the general increase in total emissions, the released compounds remain predominantly terpenes originating from the wood. This indicates that these materials can be safely used in storage, processing, and application without a significant risk of VOC release. Only selected volatile organic compounds are discussed in the text; the full list of identified VOCs is available in Table S2 (Supplementary Materials).

**Figure 12:**
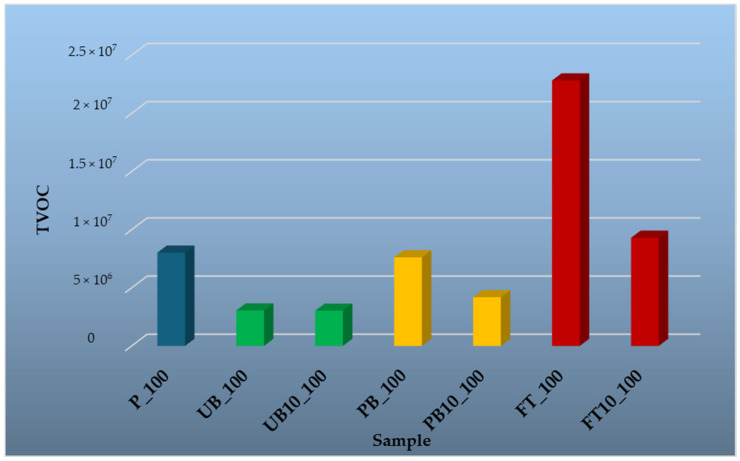
Comparison of total peak areas of volatile organic compounds (TVOC) emitted from waste automobile plastics, reference particleboard and particleboards containing plastic filler at a temperature of 100 °C.

Figure 12 provides a summary analysis of volatile organic compounds (VOCs) released at an elevated temperature of 100 °C for the various tested materials. The results clearly confirm temperature-dependent VOC emission, with the highest levels recorded in the FT_100 sample. This finding indicates that polymeric materials release significant amounts of volatile substances at higher temperatures.

In the PB_100 sample, the dominant compounds were 2,3,6-trimethyltetradecane (13.15%), 2,3,5,8-tetramethyldecane (4.88%), and 1-iodotridecane (8.21%), which is used as an intermediate in synthesis processes, for example, to construct long carbon chains in polymers [73]. In the UB_100 sample, the most abundant substances were p-xylene (5.55%) and 1-iodotridecane (5.34%). The FT_100 sample showed higher proportions of compounds such as 1,2,3-trimethylbenzene (2.47%), 3-methyldecane (2.34%), and 1-ethyl-2,3-dimethylbenzene (2.43%). In addition to these main compounds, a number of other VOCs were identified. While not all are listed in the main text, a full list of detected substances is provided in Table S3 (Supplementary Materials).

The reference particleboard (P_100) exhibited high levels of α-pinene (59.62%), followed by 3-carene (9.84%) and hexanal (4.91%). Hexanal is formed through lipid oxidation and may originate from wood resins [74]. For comparison, Jiang et al. (2017) [75] investigated the emission characteristics of formaldehyde (HCHO) and other VOCs from particleboards at different temperatures (23 °C, 35 °C, and 50 °C), with a focus on odor-active compounds. Hexanal and pentanal were identified as key odor contributors, and their concentrations increased significantly with rising temperature. However, after thermal treatment, emissions of these compounds were effectively reduced, resulting in a notable decrease in odor release at room temperature.

Particleboards containing plastic additives (PB10_100, UB10_100, FT10_100) once again predominantly emitted wood-derived compounds such as α-pinene, with proportions of 48.42% in PB10_100, 39.11% in UB10_100, and 40.2% in FT10_100. Substances detected both in pure plastic samples and in wood–plastic composites included p-xylene, o-xylene, and toluene—though toluene was also present in the reference board.

Based on these results, it can be concluded that at 100 °C, VOCs released from wooden materials are primarily monoterpenes (α-pinene, β-pinene, 3-carene, limonene), plastic materials generate aromatic hydrocarbons and alkanes (benzenes, decanes, xylenes), and composite materials containing wood and recycled polymers exhibit lower overall VOC emissions compared to pure plastics. This indicates that the wood component and plastics interact, which leads to a decrease in VOC emissions from materials prepared in this way. Similar results were also recorded by Čabalová et al. [43], who recorded a decrease in VOC emissions from single-layer particleboards with different contents of granulate from waste rubber and granulate from waste tire. It is likely that plastics are used in particleboard for their sorption properties. For example, Montes et al. [76] found that sorption of α-pinene by materials such as rubber tires, silicone rubber and Kraton was very rapid, with more than 90% of α-pinene absorbed after 15 min. These findings support the hypothesis that recycled plastics as an additive to particleboard do not contribute to increased VOC emissions to the environment and in the indoor environment of buildings.

**Figure 13:**
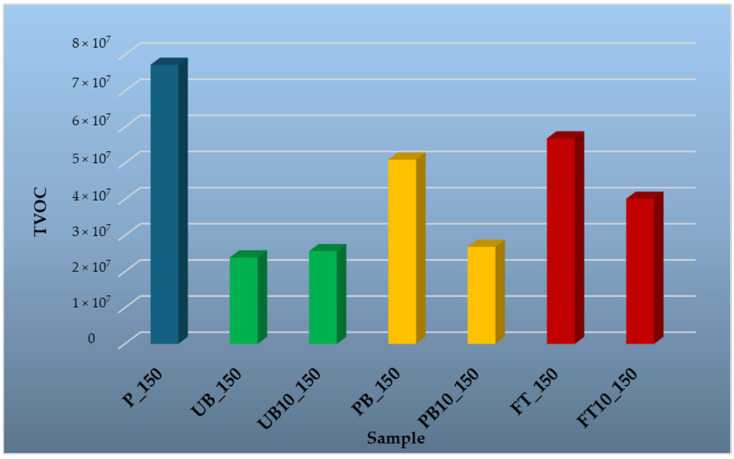
Comparison of total peak areas of volatile organic compounds (TVOC) emitted from waste automobile plastics, reference particleboard and particleboards containing plastic filler at a temperature of 150 °C.

Figure 13 presents an analysis of volatile organic compounds (VOCs) released at an elevated temperature of 150 °C from various tested materials. At this temperature, the highest VOC emissions were observed in the P_150 sample, with dominant compounds being monoterpenes such as α-pinene (13.02%), β-pinene (3.29%), and 3-carene (2.33%), primarily derived from the wood component. α-pinene is one of the main volatile organic compounds emitted into the air by wood processing industries. It is an irritant, which irritates the eyes, skin, and respiratory tract [76]. Therefore, the significant reduction in VOC emissions at 150 °C (Figure 13) by replacing 10% of the wood in particleboard with plastic is positive news. In addition to monoterpenes, sample P_150 also emitted toluene (0.71%), furfural (1.14%), and methenamine (9.27%). Furfural is mainly formed as a result of thermal degradation of hemicelluloses in wood [77], while toluene may originate from lignin degradation or adhesives used in the boards. Toluene was the dominant compound among the aromatic hydrocarbons, likely formed through deoxygenation of 4-methylguaiacol and dehydroxylation of 4-methylphenol [78].

In the PB_150 plastic sample, the most abundant compounds were 1-iodotridecane (8.22%), 2,3,6-trimethyltetradecane (7.81%), and α-methylstyrene (4.13%), which, however, were not subsequently detected in the particleboard containing this plastic. In contrast, the UB_150 sample was dominated by compounds such as butylated hydroxytoluene (8.33%), 1-iodotridecane (5.64%), and 5,5,7,7-tetraethylundecane (3.74%). For the FT_150 sample, the main compounds included nonadecane (3.54%), eicosane (2.57%), and 6-methyltridecane (1.98%).

In composite samples containing 10% plastic components, partial suppression of VOC emissions from polymers was observed, probably as a result of interactions between individual components of the composite. The dominant compounds across these samples were methenamine, α-pinene, benzaldehyde, and hexanal, which are characteristic primarily of the wood matrix. Methenamine can result from chemical interactions between formaldehyde (from adhesives) and nitrogenous compounds present in the wood [79].

In the PB10_150 sample, the dominant compound was methenamine (19.34%), followed by α-pinene (8.55%) and benzaldehyde (5.95%). The UB10_150 sample showed similar composition, with methenamine (17.56%), α-pinene (11.38%), and benzaldehyde (3.77%). In the FT10_150 sample, the most abundant compounds were methenamine (16.38%), α-pinene (13.24%), and benzaldehyde (4.03%). These results highlight the significant influence of the wood component on the overall VOC emission profile, even at elevated temperatures, as well as the differences between individual plastic additives and their interactions with the particleboard matrix. Only selected volatile organic compounds are discussed in the text; the full list of identified VOCs is available in Table S4 (Supplementary Materials).

Softwoods emit the highest concentrations of wood-derived VOCs, primarily consisting of volatile terpenes (70–90%), with lower amounts of hexanal and acetic acid (10–25%). However, thermal treatment significantly reduces total VOC emissions from softwoods by drastically decreasing volatile terpenes and hexanal while increasing acetic acid and furfural emissions [80]. Terpenes, benzenes, and derivative esters are also identified as the main components of wood and wood extracts [81].

In addition to natural wood emissions, VOCs are also released from recycled and engineered materials. Fuller et al. [82] optimized the HS-SPME/GC-MS method to analyze VOC emissions from recycled plastic resin (85–90% PP, 10–15% HDPE) and identified aliphatic hydrocarbons and 13 additive residues. The primary detected compounds included 2,4-dimethyl-heptane, 4-methyl-octane, and octamethylcyclotetrasiloxane, while D-limonene, likely originating from fragrance and flavor additives, was the most prominent peak. Other detected compounds included paraffins (C11–C15), styrene (a monomer of polystyrene), and various residues from household product additives, suggesting that significant amounts of substances from previous use, as well as polymer degradation products, remain in recycled plastics.

Similarly, engineered wood products and construction materials contribute to indoor VOC emissions. Kang et al. [83] investigated VOC emissions from low-VOC adhesive-bonded materials using small chamber tests and field measurements in residential units. They found that emissions from a wallpaper composite (PVC wallpaper + paper adhesive + gypsum board) were higher in real-world conditions than in laboratory tests, likely due to sorption effects. Toluene was the primary emitted compound from flooring composites and, to a lesser extent, from wallpaper, while m,p-xylene, ethylbenzene, and o-xylene were also detected in flooring materials. The results indicated that wallpaper can adsorb VOCs from other building materials and later re-emit them into indoor air, with toluene emissions increasing at higher temperatures.

Kim et al. [63] further analyzed VOC emissions from particleboards, medium-density fiberboard (MDF), and wood-based flooring using a test chamber and GC-MS measurements. They found that the particleboards emitted high levels of hexanal, pinenes, pentanal, nonanal, heptanal, and octanol, while wood flooring released additional compounds such as methyl acetate, vinyl acetate, toluene, methyl butyrate, and copaene.

These findings highlight the diverse sources of VOC emissions from both natural and processed materials, emphasizing the need to consider both synthetic and naturally occurring VOCs when assessing air quality. Despite rising temperatures and the potential for higher VOC emissions [82,83], composite materials containing both wood and plastic effectively limit the release of volatile organic compounds derived from both polymers and wood particles, thereby enhancing the environmental potential of these materials and their suitability for applications exposed to elevated temperatures.

## 4. Conclusions

This study investigated the impact of plastic fillers derived from automotive waste (painted and unpainted bumpers, fuel tanks) on the mechanical, physical, and chemical properties of three-layer particleboards (P). The plastic filler was incorporated into the core layer at a content of 10% by volume, with an assessment of its effects on physical properties, mechanical properties, and the emission of volatile organic compounds (VOCs). The results demonstrated that adding plastic waste to particleboards has the potential to enhance certain material properties while also enabling the efficient utilization of recycled plastics from the automotive industry.

Regarding physical properties, no significant differences were observed in water absorption (WA) and thickness swelling (TS) after 2 and 24 h compared to the reference (P) without plastic filler. This result suggests that a 10% proportion of plastic particles does not significantly affect the board’s water absorption capacity. This is likely due to the relatively low plastic concentration, which is insufficient to eliminate the hydrophilic nature of wood fibers. If the plastic content were higher, the wood fibers would be replaced by hydrophobic plastic particles to a greater extent, potentially reducing water absorption. From an application perspective, this means that these composites retain their original properties and can be used in environments with moderate moisture exposure, such as indoor applications where some level of moisture resistance is required.

Mechanical tests showed that the modulus of elasticity (MOE) and modulus of rupture (MOR) did not exhibit significant differences between the reference and plastic-filled boards. This indicates that bending strength remains stable even with the addition of plastic waste, likely due to the higher density of plastic particles, which help maintain mechanical strength. On the other hand, internal bond strength (IB) decreased when painted plastic bumpers were used, whereas unpainted bumpers did not show a significant deterioration in this parameter. This suggests that surface treatments on plastics, such as paints and coatings, may interfere with adhesion between the plastic and adhesive, weakening the bond in the core layer. Therefore, when using recycled plastics in particleboards, it is preferable to use unpainted fillers, which provide better adhesive properties and higher structural integrity.

Screw driving torque was assessed through seating torque (SeT) and stripping torque (StT) tests. The results indicated a slight decrease in SeT values for boards containing unpainted bumpers and fuel tanks, while StT remained stable across all groups. This means that the material’s ability to resist screw withdrawal was not negatively affected, which is a crucial factor in applications requiring reliable assembly. The slight reduction in SeT may be due to changes in the density distribution of the core layer, where plastic particles may slightly affect resistance to screw insertion. Nevertheless, these boards meet the required mechanical fastening standards and can be used in structural applications where good fastening capability is necessary, such as in furniture manufacturing or interior paneling.

The analysis of VOC emissions revealed that the dominant compounds released from plastic-filled particleboards were mainly monoterpenes such as α-pinene, β-pinene and 3-carene, which originate from the wood component. The key finding was that the addition of automotive plastic waste did not lead to an increase in total VOC emissions and in many cases even partially suppressed VOC emissions. In practical terms, this means that particleboards with a 10% plastic content could be used indoors without any significant risk of the plastic negatively affecting the VOC composition and increasing total emissions.

Among the three tested particleboard variants containing plastic fillers, the best properties were observed in boards with unpainted bumpers (UB10). These boards achieved the best balance between mechanical strength, screw driving torque, and environmental safety while maintaining good adhesion in the core layer. Boards with painted bumpers exhibited a slight reduction in internal bonding, suggesting that surface treatments might negatively impact bond quality. (P) containing fuel tanks also demonstrated stable performance, but they did not offer significant advantages over unpainted bumper-filled boards.

Based on these findings, it can be concluded that incorporating recycled plastics from the automotive industry into three-layer particleboards is an effective solution that reduces environmental impact while maintaining standard mechanical and physical properties. These boards are suitable for a wide range of applications, including furniture production, interior wall panels, structural elements, and decorative surfaces. Given their stable mechanical properties and good screw driving torque, they can also be used in construction and interior design applications where a combination of strength, dimensional stability, and environmental sustainability is required.

Overall, the results indicate that three-layer particleboards with the addition of 10% recycled plastic present a viable alternative to standard particleboards, providing a sustainable solution for material recovery of plastic waste from the automotive sector.

## Figures and Tables

**Table 1:** Composition and material quantities used for the production of one three-layer particleboard.

Layer	Wood Particles (g)	Resin (g)	Paraffin Emulsion (g)	Hardener (g)	Plastic Granulate (g)
Surface Up	335	51.95	7.25	2.2	0
Core layer	888.3	99.1	16.3	8.4	98.7
Surface Down	335	51.95	7.25	2.2	0

**Table 2:** Designations of particleboard type.

Signification	Composite Characteristics
P	Reference three-layer particleboard without plastic filler
FT10	Three-layer particleboard with 10% fuel tanks in core layer
PB10	Three-layer particleboard with 10% painted bumpers in core layer
UB10	Three-layer particleboard with 10% unpainted bumpers in core layer

**Table 3:** List of sample abbreviations and material types with GC-MS testing temperatures.

Sample	Explanation of the Designation
P_35; P_50; P_100; P_150	Reference particleboard, VOC analysis at 35, 50, 100, and 150 °C
UB_35; UB_50; UB_100; UB_150	Unpainted bumpers, VOC analysis at 35, 50, 100, and 150 °C
UB10_35; UB10_50; UB10_100; UB10_150	Particleboard with 10% unpainted bumpers content in the core layer, VOC analysis 35, 50, 100, and 150 °C
PB_35; PB_50; PB_100; PB_150	Painted bumpers, VOC analysis at 35, 50, 100, and 150 °C
PB10_35; PB10_50; PB10_100; PB10_150	Particleboard with 10% painted bumpers content in the core layer, VOC analysis at 35, 50, 100, and 150 °C
FT_35; FT_50; FT_100; FT_150	Fuel tanks, VOC analysis at 35, 50, 100, and 150 °C
FT10_35; FT10_50; FT10_100; FT10_150	Particleboard with 10% fuel tanks content in the core layer, VOC analysis at 35, 50, 100, and 150 °C

**Table 4:** Average values of density.

Sample	Density (g·cm^−3^)
P	0.72 ± 0.008
FT10	0.72 ± 0.029
PB10	0.70 ± 0.023
UB10	0.71 ± 0.031

Explanation of abbreviations: P—reference particleboard; FT10—particleboard containing 10% waste fuel tanks; PB10—particleboard containing 10% waste painted bumpers; UB10—particleboard containing 10% waste unpainted bumpers.

**Table 5:** Average values of physical properties and ANOVA analysis results.

Composite Sample	Water Absorption (%)		Thickness Swelling (%)	
	2 h	24 h	2 h	24 h
P	12.8 * ± 1.85	40.0 * ± 5.35	5.6 * ± 0.86	18.0 * ± 2.87
FT10	12.7 * ± 2.15	41.9 * ± 7.53	4.7 * ± 0.20	16.8 * ± 2.26
PB10	11.8 * ± 1.78	41.6 * ± 10.05	5.1 * ± 1.15	18.1 * ± 3.64
UB10	10.8 * ± 1.38	36.9 * ± 4.78	5.5 * ± 1.37	16.8 * ± 1.73
F	1.831	0.754	1.025	0.505
Sig.	0.168	0.531	0.399	0.683

*p* ≤ 0.05; *: No significant difference was found. Explanation of abbreviations: P—reference particleboard; FT10—particleboard containing 10% waste fuel tanks; PB10—particleboard containing 10% waste painted bumpers; UB10—particleboard containing 10% waste unpainted bumpers.

**Table 6:** Average values of mechanical properties.

Composite Sample	IB (MPa)	MOR (MPa)	MOE (MPa)	SDT (N·m)	
				SeT	StT
P	0.52 ^a^ ± 0.05	13.06 ^a^ ± 1.25	2294.46 * ± 275.44	0.45 ^a^ ± 0.04	1.45 * ± 0.06
FT10	0.42 ^b^ ± 0.05	11.96 ^ab^ ± 1.47	2203.88 * ± 146.27	0.37 ^b^ ± 0.03	1.31 * ± 0.14
PB10	0.39 ^b^ ± 0.05	12.63 ^a^ ± 1.11	2239.91 * ± 207.33	0.37 ^b^ ± 0.03	1.28 * ± 0.06
UB10	0.48 ^a^ ± 0.05	10.50 ^b^ ± 1.27	2056.28 * ± 199.57	0.42 ^ab^ ± 0.08	1.30 * ± 0.14
F	9.313	4.314	1.505	3.743	2.072
Sig.	0.000	0.017	0.244	0.024	0.131

Note: Different letters indicate different homogeneity groups, *p* ≤ 0.05; *: No significant difference was found. Explanation of abbreviations: IB—internal bonding strength; MOR—modulus of rupture; MOE—modulus of elasticity; P—reference particleboard; FT10—particleboard containing 10% waste fuel tanks; PB10—particleboard containing 10% waste painted bumpers; UB10—particleboard containing 10% waste unpainted bumpers.

## Data Availability

The data presented in this study are available on request from the corresponding author.

## Supplementary Materials

The following supporting information can be downloaded at: https://www.mdpi.com/article/10.3390/polym17111438/s1, Table S1: VOCs identified by GC–MS at 35 °C with corresponding retention times and their percentage representation in the total chromatographic peak area; Table S2: VOCs identified by GC–MS at 50 °C with corresponding retention times and their percentage representation in the total chromatographic peak area; Table S3: VOCs identified by GC–MS at 100 °C with corresponding retention times and their percentage representation in the total chromatographic peak area; Table S4: VOCs identified by GC–MS at 150 °C with corresponding retention times and their percentage representation in the total chromatographic peak area.

## Abbreviations

The following abbreviations are used in this manuscript:

P	Reference Particleboard
UB	Unpainted Bumpers
PB	Painted Bumpers
FT	Fuel Tanks
UF	Urea—Formaldehyde
HDPE	High Density Polyethylene
PP	Polypropylene
UB10	Particleboard Containing 10% Unpainted Bumpers
PB10	Particleboard Containing 10% Painted Bumpers
FT10	Particleboard Containing 10% Fuel Tanks
WA	Water Absorption
TS	Thickness Swelling
IBS	Internal Bonding Strength
BS	Bonding Strength
MOR	Modulus of Rupture
MOE	Modulus of Elasticity
SDT	Screw Driving Torque
SeT	Seating Torque
StT	Stripping Torque
VOC	Volatile Organic Compound
GC-MS	Gas Chromatography—Mass Spectrometry
MAPP	Maleic Anhydride-Modified Polypropylene
MDF	Middle Density Fiberboard

## References

[1] Thoemen H., Irle M., Sernek M. (2010). Wood-Based Panels: An Introduction for Specialists.

[2] Forest Products Laboratory (1999). Wood Handbook: Wood as an Engineering Material.

[3] Kurşun C., Onat S., Kelleci O. (2023). Particleboard Density and Surface Quality. Proceedings of the 2nd International Conference on Contemporary Academic Research.

[4] Pothan L.A., Luyt A.S., Thomas S., Nwabunma D., Kyu T. (2007). Polyolefin/Natural Fiber Composites. Polyolefin Composites.

[5] Klyosov A. (2007). Wood-Plastic Composites.

[6] Carus M., Eder A., Dammer L., Korte H., Scholz L., Essel R., Breitmayer E., Barth M. (2015). Wood-Plastic Composites (WPC) and Natural Fibre Composites (NFC): European and Global Markets 2012 and Future Trends in Automotive and Construction.

[7] Clemons C. (2002). Wood-Plastic Composites in the United States: The Interfacing of Two Industries. For. Prod. J..

[8] Meikle J.L. (1997). Material Doubts: The Consequences of Plastic. Environ. Hist..

[9] Haque M., Goda K., Ito H., Ogoe S., Okamot M., Ema T., Kagawa K., Nogami H. (2018). Melt-Viscosity and Mechanical Behaviour of Polypropylene (PP)/Wood Flour Composites: Effect of Pulverization of Wood Flour with and without Water. Adv. Ind. Eng. Polym. Res..

[10] Gogoi R., Manik G., Mavinkere Rangappa S., Parameswaranpillai J., Kumar M.H., Siengchin S. (2021). Mechanical Properties of Wood Polymer Composites.

[11] Kazemi Najafi S. (2013). Use of Recycled Plastics in Wood Plastic Composites—A Review. Waste Manag..

[12] Wolcott M.P., Buschow K.H.J., Cahn R.W., Flemings M.C., Ilschner B., Kramer E.J., Mahajan S., Veyssière P. (2001). Wood–Plastic Composites. Encyclopedia of Materials: Science and Technology.

[13] Rowell R.M. (2012). Handbook of Wood Chemistry and Wood Composites.

[14] Jambrekovic V., Antonovic A., Kljak J., Brezovic M. (2005). The Applicability of Wood and Plastic Composites in Construction. Wood in the Construction Industry: Durability and Quality of Wooden Construction Products, Proceedings of the 7th International Conference, Zagreb, Croatia, 22 April 2005.

[15] Xu H., Yang Y., Li L., Liu B., Fu X., Yang X., Cao Y. (2023). Mechanical Properties Variation in Wood—Plastic Composites with a Mixed Wood Fiber Size. Materials.

[16] Khamedi R., Hajikhani M., Ahmaditabar K. (2019). Investigation of Maleic Anhydride Effect on Wood Plastic Composites Behavior. J. Compos. Mater..

[17] Selke S.E., Wichman I. (2004). Wood Fiber/Polyolefin Composites. Compos. Part A Appl. Sci. Manuf..

[18] Lei Y., Wu Q., Yao F., Xu Y. (2007). Preparation and Properties of Recycled HDPE/Natural Fiber Composites. Compos. Part A Appl. Sci. Manuf..

[19] Adhikary K.B., Pang S., Staiger M.P. (2008). Dimensional Stability and Mechanical Behaviour of Wood–Plastic Composites Based on Recycled and Virgin High-Density Polyethylene (HDPE). Compos. Part B Eng..

[20] Najafi S.K., Hamidinia E., Tajvidi M. (2006). Mechanical Properties of Composites from Sawdust and Recycled Plastics. J. Appl. Polym. Sci..

[21] Jayaraman K., Halliwell R. (2009). *Harakeke* (*Phormium Tenax*) Fibre–Waste Plastics Blend Composites Processed by Screwless Extrusion. Compos. Part B Eng..

[22] Salthammer T., Mentese S., Marutzky R. (2010). Formaldehyde in the Indoor Environment. Chem. Rev..

[23] Roffael E. (2006). Volatile Organic Compounds and Formaldehyde in Nature, Wood and Wood Based Panels. Holz Als Roh-Werkstoff.

[24] Adamová T., Hradecký J., Pánek M. (2020). Volatile Organic Compounds (VOCs) from Wood and Wood-Based Panels: Methods for Evaluation, Potential Health Risks, and Mitigation. Polymers.

[25] Hodgson A., Beal D., McIlvaine J.E.R. (2003). Sources of Formaldehyde, Other Aldehydes and Terpenes in a New Manufactured House. Indoor Air.

[26] Felix J.S., Domeno C., Nerin C. (2013). Characterization of Wood Plastic Composites Made from Landfill-Derived Plastic and Sawdust: Volatile Compounds and Olfactometric Analysis. Waste Manag..

[27] Vaisanen T., Laitinen K., Tomppo L., Joutsensaari J., Raatikainen O., Lappalainen R., Yli-Pirila P. (2018). A Rapid Technique for Monitoring Volatile Organic Compound Emissions from Wood-Plastic Composites. Indoor Built Environ..

[28] Benthien J.T., Ohlmeyer M. (2013). Thickness Swelling and Water Absorption of WPC after Immersion in Cold and Boiling Water. Eur. J. Wood Prod..

[29] Tenorio C., Roque R., Camacho D. (2012). Physical and Mechanical Properties of Plywood Panels Manufactures with Tropical Plantaion Species for Structural Use. CERNE.

[30] Mancel V., Čabalová I., Krilek J., Olgun Ç., Öncel M., Tor O., Szul T., Woroniak G., Woroniak J. (2025). Utilization of Waste Rubber Materials After the End of Their Life Cycle in the Production of Three-Layer Particleboards—Physical and Mechanical Properties. Polymers.

[31] Xu J., Sugawara R., Widyorini R., Han G., Kawai S. (2004). Manufacture and Properties of Low-Density Binderless Particleboard from Kenaf Core. J. Wood Sci..

[32] Tung C.Y., Wang M.H. (2002). Comparison of Environmental Performance between Plastic and Steel Fuel Tanks. Proc. Inst. Mech. Eng. Part B-J. Eng. Manuf..

[33] Benrabah Z., Thibault F., DiRaddo R. (2011). Modeling of Fuel Permeation in Multilayer Automotive Plastic Fuel Tanks. SAE Int. J. Mater. Manuf..

[34] Alvarado P.J. (1996). Steel vs Plastics: The Competition for Light-Vehicle Fuel Tanks. JOM.

[35] Cho D.L., Shin K.H., Lee W.J., Kim D.H. (2001). Improvement of Paint Adhesion to a Polypropylene Bumper by Plasma Treatment. J. Adhes. Sci. Technol..

[36] Zhang H., Zheng H. (2021). Artificial and Natural Aging of Polypropylene Used in Passenger Vehicle Bumpers. Mater. Plast..

[37] Rajmohan K.V.S., Ramya C., Raja Viswanathan M., Varjani S. (2019). Plastic Pollutants: Effective Waste Management for Pollution Control and Abatement. Curr. Opin. Environ. Sci. Health.

[38] Wright S.L., Kelly F.J. (2017). Plastic and Human Health: A Micro Issue?. Environ. Sci. Technol..

[39] Liu E.K., He W.Q., Yan C.R. (2014). ‘White Revolution’ to ‘White Pollution’—Agricultural Plastic Film Mulch in China. Environ. Res. Lett..

[40] Zhang X., Yu J., Yan W., Wang Y., Subramanian N. (2023). A Comprehensive Review of Reverse Logistics in the Automotive Industry. IEEE Access.

[41] Krilek J., Čabalová I., Mancel V., Réh R. Three-Layer Particleboard with Added Rubber. Utility Model No. 10249.2025. (In Slovak). https://wbr.indprop.gov.sk/WebRegistre/UzitkovyVzor/Detail/81-2024?HlaskaStav=VyhodHlasku&ItemPPType=NotSelected&SearchType=NoSearch&Lang=en&SearchResultsMaxReached=False&HighlightSearchCriteria=True&SearchExternalDatabases=False&IsFulltextSearch=False&SearchTimeout=False&SearchCBO_PoslednyRokPlatnosti=False&SearchCBO_AktualnaPlatnostKonciNasledujuciMesiac=False&IsSearchSimilarMPT=True&IsSearchSimilarMTD=True&IsSearchSimilarMTOP=True&IsSearchSimilarPrihlasovatelovMajitelov=True&IsSearchSimilarPovodcov=True&IsSearchSimilarZnenieReprodukciuOZ=True&IsSearchSimilarCisloZakladnehoPatentu=True&csrt=18118162609265738128.

[42] Mancel V., Krilek J., Čabalová I., Réh R., Osvaldová M., Darabošová A. (2024). Evaluation of selected mechanical and physical properties of particleboards containing waste plastics. Wood Res..

[43] Čabalová I., Krilek J., Bubenikova T., Ružiak I., Němec M., Lee S.H., Lubis M.A., Darabošová A., Mancel V., Kristak L. (2025). Utilization of Waste Tire and Rubber from Automobiles in the Manufacturing of Particleboards and Evaluation of Its Properties. Eur. J. Wood Wood Prod..

[44] Čabalová I., Ház A., Krilek J., Bubeníková T., Melicherčík J., Kuvik T. (2021). Recycling of Wastes Plastics and Tires from Automotive Industry. Polymers.

[45] (2017). Standard Test Methods for Density and Specific Gravity (Relative Density) of Wood and Wood-Based Materials.

[46] (2020). Standard Test Methods for Evaluating Properties of Wood-Base Fiber and Particle Panel Materials.

[47] Požgaj A., Chovanec D., Kurjatko S., Babiak M. (1997). Štruktúra a Vlastnosti Dreva.

[48] Deppe H.-J., Ernst K. (1991). Taschenbuch Der Spanplattentechnik.

[49] Piringer O.G., Baner A.L. (2008). Plastic Packaging Materials for Food: Barrier Function, Mass Transport, Quality Assurance, and Legislation.

[50] Gozdecki C., Wilczyński A., Kociszewski M., Zajchowski S. (2015). Properties of Wood–Plastic Composites Made of Milled Particleboard and Polypropylene. Eur. J. Wood Prod..

[51] Li B., Zheng Y., Pan Z., Hartsough B. (2009). Improved Properties of Medium-Density Particleboard Manufactured from Saline Creeping Wild Rye and HDPE Plastic. Ind. Crops Prod..

[52] Chaharmahali M., Tajvidi M., Kazemi S. (2008). Mechanical Properties of Wood Plastic Composite Panels Made from Waste Fiberboard and Particleboard. Polym. Compos..

[53] Bütün F.Y., Sauerbier P., Militz H., Mai C. (2019). The Effect of Fibreboard (MDF) Disintegration Technique on Wood Polymer Composites (WPC) Produced with Recovered Wood Particles. Compos. Part A Appl. Sci. Manuf..

[54] Kamdem P., Jiang H., Cui W., Freed J., Matuana L. (2004). Properties of Wood Plastic Composites Made of Recycled HDPE and Wood Flour from CCA-Treated Wood Removed from Service. Compos. Part A Appl. Sci. Manuf..

[55] Yu X., Tor O., Quin F., Seale D., Zhang J. (2015). Screwdriving Torques in Particleboards. Wood Fiber Sci. J. Soc. Wood Sci. Technol..

[56] Tor O., Yu X., Zhang J. (2015). Characteristics of Torques for Driving Screws into Wood-Based Composites. Wood Fiber Sci..

[57] Zhou S., Liu H., Ding Y., Wu Y. (2019). The Effects of Temperature and Humidity on the VOC Emission Rate from Dry Building Materials. IOP Conf. Ser. Mater. Sci. Eng..

[58] Zhou Y., Wu J. (2012). Thermodynamic Properties of O-Xylene, m-Xylene, p-Xylene, and Ethylbenzene. J. Phys. Chem. Ref. Data.

[59] Danon B., van der Gryp P., Schwarz C.E., Görgens J.F. (2015). A Review of Dipentene (Dl-Limonene) Production from Waste Tire Pyrolysis. J. Anal. Appl. Pyrolysis.

[60] Pakdel H., Pantea D.M., Roy C. (2001). Production of *Dl*-Limonene by Vacuum Pyrolysis of Used Tires. J. Anal. Appl. Pyrolysis.

[61] Honnet S., Seshadri K., Niemann U., Peters N. (2009). A Surrogate Fuel for Kerosene. Proc. Combust. Inst..

[62] Romanenko E.P., Tkachev A.V. (2006). Identification by GC–MS of Cymene Isomers and 3,7,7-Trimethylcyclohepta-1,3,5-Triene in Essential Oils. Chem. Nat. Compd..

[63] Kim S., Kim J.-A., Kim H.-J., Kim S.-D. (2006). Determination of Formaldehyde and TVOC Emission Factor from Wood-Base Composites by Small Chamber Method. Polym. Test..

[64] Brown S. (1999). Chamber Assessment of Formaldehyde and VOC Emissions from Wood-Based Panels. Indoor Air.

[65] Yrieix C., Dulaurent A., Laffargue C., Maupetit F., Pacary T., Uhde E. (2010). Characterization of VOC and Formaldehyde Emissions from a Wood Based Panel: Results from an Inter-Laboratory Comparison. Chemosphere.

[66] Hyttinen M., Masalin-Weijo M., Kalliokoski P., Pasanen P. (2010). Comparison of VOC Emissions between Air-Dried and Heat-Treated Norway Spruce (*Picea abies*), Scots Pine (*Pinus sylvesteris*) and European Aspen (*Populus tremula*) Wood. Atmos. Environ..

[67] Judzentiene A., Kupcinskiene E. (2008). Chemical Composition on Essential Oils from Needles of *Pinus Sylvestris* L. Grown in Northern Lithuania. J. Essent. Oil Res..

[68] Guillory J. (2007). The Merck Index:  An Encyclopedia of Chemicals, Drugs, and Biologicals Edited by Maryadele J. O’Neil, Patricia E. Heckelman, Cherie B. Koch, and Kristin J. Roman. Merck, John Wiley & Sons, Inc., Hoboken, NJ. 2006. xiv + 2564 pp. 18 × 26 cm. ISBN-13 978-0-911910-001. $125.00. J. Med. Chem..

[69] Marć M. (2020). Emissions of Selected Monoaromatic Hydrocarbons as a Factor Affecting the Removal of Single-Use Polymer Barbecue and Kitchen Utensils from Everyday Use. Sci. Total Environ..

[70] Lin S.-Y., Tseng J.-M., Lee M.-K., Wu T.-C., Shu C.-M. (2009). Thermal Runaway Evaluation of α-Methylstyrene and Trans-β-Methylstyrene with Benzaldehyde. J. Therm. Anal. Calorim..

[71] Jia C., Batterman S. (2010). A Critical Review of Naphthalene Sources and Exposures Relevant to Indoor and Outdoor Air. Int. J. Environ. Res. Public Health.

[72] Cui Y., Qu Z., Bao W., Lv Y. (2016). Study on Methenamine Detection in Starch Products through SERS Technology. Selected Proceedings of the Chinese Society for Optical Engineering Conferences.

[73] Buy 1-Iodotridecane|35599-77-0|BenchChem. https://www.benchchem.com/product/b1606830.

[74] Sitbon C., Paliyath G., Moo-Young M. (2011). 4.28—Pre- and Postharvest Treatments Affecting Nutritional Quality. Comprehensive Biotechnology.

[75] Jiang C., Li D., Zhang P., Li J., Wang J., Yu J. (2017). Formaldehyde and Volatile Organic Compound (VOC) Emissions from Particleboard: Identification of Odorous Compounds and Effects of Heat Treatment. Build. Environ..

[76] Montes M., Daugulis A.J., Veiga M.C., Kennes C. (2011). Characterization of Absorbent Polymers for the Removal of Volatile Hydrophobic Pollutants from Air. J. Chem. Technol. Biotechnol..

[77] Luo Y., Li Z., Li X., Liu X., Fan J., Clark J.H., Hu C. (2019). The Production of Furfural Directly from Hemicellulose in Lignocellulosic Biomass: A Review. Catal. Today.

[78] Zhao J., Wang X., Hu J., Liu Q., Shen D., Xiao R. (2014). Thermal Degradation of Softwood Lignin and Hardwood Lignin by TG-FTIR and Py-GC/MS. Polym. Degrad. Stabil..

[79] Van Den Velde S., Van Steenberghe D., Van Hee P., Quirynen M. (2009). Detection of Odorous Compounds in Breath. J. Dent. Res..

[80] Pohleven J., Burnard M.D., Kutnar A. (2019). Volatile Organic Compounds Emitted from Untreated and Thermally Modified Wood-a Review. Wood Fiber Sci..

[81] Gierlinger N., Jacques D., Schwanninger M., Wimmer R., Pâques L. (2004). Heartwood Extractives and Lignin Content of Different Larch Species (*Larix* sp.) and Relationships to Brown-Rot Decay-Resistance. Trees.

[82] Fuller J., White D., Yi H., Colley J., Vickery Z., Liu S. (2020). Analysis of Volatile Compounds Causing Undesirable Odors in a Polypropylene—High-Density Polyethylene Recycled Plastic Resin with Solid-Phase Microextraction. Chemosphere.

[83] Kang D.H., Choi D.H., Yeo M.S., Kim K.W. (2013). Evaluation of VOC Emission and Sorption Characteristics of Low-VOC Adhesive-Bonded Building Materials. J. Adhes. Sci. Technol..

