# Supplementary material for: Mechanical Properties, Physical Properties and VOC Emissions of Three-Layer Particleboards with Recycled Automotive Plastics in the Core Layer
Source: Polymers (Basel). 2025 May 22;17(11):1438. doi: 10.3390/polym17111438 (PMC12157565; doi:10.3390/polym17111438)
Supplement: Supplementary file 1 [file polymers-17-01438-s001.zip › polymers-3597724-supplementary.pdf]

Table S1-4. Identified substances.

Table S1. VOCs identified by GC–MS at 35°C with corresponding retention times and their percentage representation in the total chromatographic peak area.

VOC	Retention time (min)	PB_35 %	UB_35 %	FT_35 %	P_35 %	PB10_35 %	UB10_35 %	FT10_35 %
p-Xylene	6.238	---	7.52	2.09	---	---	---	---
o-Xylene	6.847	4.4	---	---	---	---	---	---
α-Pinene	7.974	---	---	---	11.04	11.13	9.5	21.37
β-Pinene	9.207	---	---	---	2.89	---	---	5.77
Benzene, 1,2,3-trimethyl-	9.694	4.92	---	5.66	---	---	---	---
3-Carene	10.196	---	---	---	1.6	5.25	---	---
Benzene, 1,2,4-trimethyl-	10.547	---	---	4.95	---	---	---	---
o-Cymene	10.622	---	---	3.43	---	---	---	---
Benzene, 1-methyl-3-propyl-	11.414	---	---	2.03	---	---	---	---
2,3,6-Trimethyltetradecane	11.612	10.05	---	---	---	---	---	---
Benzene, 2-ethyl-1,4-dimethyl-	11.627	---	---	1.91	---	---	---	---
Benzene, 1-ethyl-3,5-dimethyl-	12.175	---	---	2.23	---	---	---	---
Cyclopentene,3,5-dimethylene-1,4,4-trimethyl-	12.419	---	---	3.02	---	---	---	---
2,3-Trimethylenenorbornane	12.647	---	---	1.93	---	---	---	---
Decane, 2,3,5,8-tetramethyl-	12.936	4.33	---	---	---	---	---	---
Prehnitol	13.332	---	---	2.58	---	---	---	---
Naphthalene, decahydro-2-methyl-	13.621	---	---	3.43	---	---	---	---

Table S2. VOCs identified by GC–MS at 50°C with corresponding retention times and their percentage representation in the total chromatographic peak area.

VOC	Retention time (min)	PB_50 %	UB_50 %	FT_50 %	P_50 %	PB10_50 %	UB10_50 %	FT10_50 %
Toulene	4.001	1.03	---	0.51	---	---	---	---
p-Xylene	6.238	3.81	9.69	2.09	---	---	---	---
Styrene	6.787	0.74	---	---	---	---	---	---
o-Xylene	6.847	1.27	---	---	---	---	---	---
Ethylbenzene	6.848	---	3.71	---	---	---	---	---
α-Pinene	7.974	---	---	---	53.12	29.78	13.97	46.17
Mesitylene	8.963	0.99	---	---	---	---	---	---
β-Pinene	9.207	---	---	---	9.84	3.89	---	5.41
α-Methylstyrene	9.359	1.54	---	---	---	---	---	---
Benzene, 1,2,3-trimethyl-	9.694	4.96	---	5.34	---	---	---	---
3-Carene	10.196	---	---	---	5.2	11.0	---	---
Benzene, 1,2,4-trimethyl-	10.547	---	---	4.02	---	---	---	---
Benzene, 1-methyl-3-propyl-	11.414	---	---	1.95	---	---	---	---
2,3,6-Trimethyltetradecane	11.612	12.43	---	---	---	---	---	---
Benzene, 2-ethyl-1,4-dimethyl-	11.627	---	---	2.08	---	---	---	---
Decane, 3,6-dimethyl-	11.779	3.12	---	---	---	---	---	---
Benzene, 1-ethyl-3,5-dimethyl-	12.175	---	---	3.93	---	---	---	---
Cyclopentene,3,5-dimethylene-1,4,4-trimethyl-	12.419	---	---	2.96	---	---	---	---
2,3-Trimethylenenorbornane	12.647	---	---	1.94	---	---	---	---
Decane, 2,3,5,8-tetramethyl-	12.936	4.03	---	---	---	---	---	---
Benzene, 1-ethyl-2,3-dimethyl-	13.317	---	---	2.7	---	---	---	---
Benzene, 1,2,4,5-tetramethyl-	13.454	---	---	3.66	---	---	---	---
Naphthalene, decahydro-2-methyl-	13.621	---	---	1.47	---	---	---	---
1-Methyl-2-phenylcyclopropane	13.987	---	---	1.46	---	---	---	---
Benzene, 1-ethyl-3,5-dimethyl-	14.382	---	---	1.95	---	---	---	---
Naphthalene, decahydro-2,6-dimethyl-	15.159	---	---	1.22	---	---	---	---

Naphthalene	15.28	---	---	1.46	---	---	---	---
Methenamine	16.392	---	---	1.45	---	---	---	---
1-Iodotridecane	18.005	4.09	---	---	---	---	---	---

Table S3. VOCs identified by GC–MS at 100°C with corresponding retention times and their percentage representation in the total chromatographic peak area.

VOC	Retention time (min)	PB_100 %	UB_100 %	FT_100 %	P_100 %	PB10_100 %	UB10_100 %	FT10_100 %
Pentane, 2,3,3-trimethyl-	3.758	---	---	1.0	---	---	---	---
Toulene	4.001	0.37	0.78	---	0.8	1.18	1.71	0.72
Hexanal	4.625	---	---	---	4.91	4.99	---	1.52
Heptane, 2,4-dimethyl-	5.082	1.23	---	---	---	---	---	---
Furfural	5.401	---	---	---	---	0.65	1.27	0.47
2,4-Dimethyl-1-heptene	5.538	0.59	---	---	---	---	---	---
Piperidine	5.904	0.14	---	---	---	---	---	---
2-Methylene-hexanal	6.087	0.5	---	---	---	---	---	---
p-Xylene	6.238	1.92	5.55	0.52	---	---	0.78	0.22
Styrene	6.787	0.42	---	---	---	---	---	---
o-Xylene	6.847	0.7	1.77	---	---	0.48	---	---
Ethylbenzene	6.848	0.45	---	---	---	---	---	---
γ-Terpinen	7.639	---	---	---	---	0.68	1.21	0.51
α-Phellandrene	7.791	---	---	---	---	0.88	---	0.78
α-Pinene	7.974	---	1.04	---	59.62	48.42	39.11	40.2
Camphene	8.385	---	---	---	---	1.46	1.25	1.4
Dehydrosabinene	8.552	---	---	---	---	---	1.51	0.97
Benzaldehyde	8.735	---	---	---	1.84	1.88	---	0.94
Benzene, 1-ethyl-3-methyl-	8.765	1.48	---	0.75	---	---	---	---
Mesitylene	8.963	0.84	---	0.48	---	---	---	---
p-Cymene	9.024	---	---	---	0.93	1.42	---	1.4
Sabinene	9.116	---	---	---	---	---	0.66	0.55
β-Pinene	9.207	---	---	---	5.9	2.21	2.22	21.6
Benzene, 1-ethyl-2-methyl-	9.298	0.69	---	0.51	---	---	---	---
α-Methylstyrene	9.359	1.04	---	---	---	---	---	---
2-Pentylfuran	9.648	---	---	---	2.08	1.84	1.42	0.73
Benzene, 1,2,3-trimethyl-	9.694	3.89	2.57	2.47	---	---	---	---
Octanal	9.892	---	1.69	---	---	---	---	---
Nonane, 2,6-dimethyl-	10.166	2.12	---	---	---	---	---	---
3-Carene	10.196	---	---	---	9.84	9.87	4.35	5.6
Benzene, 1,2,4-trimethyl-	10.547	---	---	2.2	---	---	---	---
o-Cymene	10.622	---	---	---	---	1.04	2.11	1.04
Limonene	10.744	---	---	---	2.12	1.86	---	1.0
Cyclohexane, 1,1-dimethyl-2-propyl-	11.14	---	---	0.65	---	---	---	---
Salicylaldehyde	11.155	---	---	---	---	---	0.37	---
Benzene, 1-methyl-3-propyl-	11.414	---	---	1.15	---	---	---	---
Naphthalene, decahydro-	11.49	---	---	1.0	---	---	---	---
2,3,6-Trimethyltetradecane	11.612	13.15	---	---	---	---	---	---
Benzene, 2-ethyl-1,4-dimethyl-	11.627	---	---	1.33	---	---	---	---
Decane, 3,6-dimethyl-	11.779	3.18	---	---	---	---	---	---
Decane, 3-methyl-	11.993	---	---	0.3	---	---	---	---
Benzene, 1-ethyl-3,5-dimethyl-	12.175	---	---	1.68	---	---	---	---
Cyclopentene,3,5-dimethylene-1,4,4-trimethyl-	12.419	---	---	2.13	---	---	---	---
p-Cymenene	12.54	---	1.66	---	---	---	---	---
4,7-Methano-1H-indene, octahydro-	12.632	---	---	1.19	---	---	---	---
2,3-Trimethylenenorbornane	12.647	1.1	---	---	---	---	0.94	---
Decane, 3-methyl-	12.86	1.22	---	2.34	---	---	---	---
Decane, 2,3,5,8-tetramethyl-	12.936	4.88	---	---	---	---	---	---
Decane, 3,7-dimethyl-	13.104	1.79	---	---	---	---	---	---
trans-Decalin, 2-methyl-	13.134	---	---	0.91	---	---	---	---

Benzene, 1-ethyl-2,3-dimethyl-	13.317	1.54	---	2.43	---	---	---	---
Benzene, 1,2,4,5-tetramethyl-	13.454	---	---	1.99	---	---	---	---
Naphthalene, decahydro-2-methyl-	13.621	---	2.15	1.9	---	---	---	---
Benzene, 1,3-diethyl-5-methyl-	14.154	---	---	0.81	---	---	---	---
L-camphor	14.185	---	---	---	---	---	---	1.58
Benzene, 1-ethenyl-4-ethyl-	14.306	---	0.77	1.54	---	---	---	---
Benzene, 1-ethyl-3,5-dimethyl-	14.382	0.3	---	2.02	---	---	---	---
Pinocarvone	14.717	---	---	---	---	---	---	0.4
Naphthalene, decahydro-2,6-dimethyl-	15.159	---	---	1.1	---	---	---	---
Naphthalene	15.28	0.23	0.55	1.0	---	---	---	---
Tridecane, 6-methyl-	15.767	---	3.1	---	---	---	---	---
Naphthalene, decahydro-2,6-dimethyl-	15.904	---	---	1.01	---	---	---	---
(1S)-Camphorquinone	16.087	---	---	---	---	0.78	---	---
Undecane, 2,6-dimethyl-	16.163	---	---	1.53	---	---	---	---
Cyclohexane, 2-butyl-1,1,3-trimethyl-	16.376	---	---	1.51	---	---	---	---
Methenamine	16.392	---	---	0.77	---	---	---	---
Dodecane, 4,6-dimethyl-	16.787	0.4	---	---	---	---	---	---
Hexadecane, 2,6,10,14-tetramethyl-	17.015	0.93	---	---	---	---	---	---
Tridecanol, 2-ethyl-2-methyl-	17.837	0.89	---	---	---	---	---	---
1-Iodotridecane	18.005	8.21	5.34	---	---	---	---	---
Undecane	18.385	---	1.31	1.43	---	---	---	---
Tridecane, 6-methyl-	18.538	---	---	1.96	---	---	---	---
Tetradecane, 1-iodo-	19.253	2.75	---	---	---	---	---	---
Dodecane, 1-iodo-	19.497	0.75	---	---	---	---	---	---
Dodecane, 2,7,10-trimethyl-	20.562	---	---	1.15	---	---	---	---
1H-Indene, 2,3-dihydro-1,1,3-trimethyl-	20.989	---	---	0.9	---	---	---	---
1,3-DIACETYLBENZENE	22.023	0.87	---	---	---	---	---	---
2,6,10-Trimethyltridecane	22.724	---	---	0.9	---	---	---	---
Benzoquinone, 2,6-di-(1,1-dimethylethyl)	22.906	---	0.59	---	---	---	---	---
Undecane, 3,8-dimethyl-	23.454	0.22	---	---	---	---	---	---
5,5,7,7-Tetraethylundecane	23.576	1.78	---	---	---	---	---	---
Butylated Hydroxytoulene	24.033	---	2.97	0.45	---	---	---	---
Octadecane, 3-ethyl-5-(2-ethylbutyl)-	24.642	0.8	---	---	---	---	---	---
Nonadecane	26.012	0.26	---	2.07	---	---	---	0.8
Crocetane	28.371	---	---	0.58	---	---	---	---
Eicosane	30.365	---	---	1.17	---	---	---	---
Heptadecane, 2,6,10,15-tetramethyl-	30.578	---	---	0.36	---	---	---	---
Heneisocane	32.405	---	---	0.81	---	---	---	---

Table S4. VOCs identified by GC–MS at 150 °C with corresponding retention times and their percentage representation in the total chromatographic peak area.

VOC	Retention time (min)	PB_150 %	UB_150 %	FT_150 %	P_150 %	PB10_150 %	UB10_150 %	FT10_150 %
Cyclohexane, methyl-	3.285	---	0.37	---	---	---	---	---
Pyrazine	3.468	0.63	---	---	---	0.35	0.4	0.28
1,4-Cyclohexadiene, 1-methyl-	3.575	---	---	0.17	---	---	---	---
Pyridine	3.727	---	---	---	0.42	0.87	0.94	0.64
Pentane, 2,3,3-trimethyl-	3.758	---	0.59	0.36	---	---	---	---
1,3,5-Hexatriene, 2-methyl-	3.849	---	---	0.08	---	---	---	---
Toulene	4.001	---	---	0.12	0.74	1.45	1.23	0.97
Hexanal	4.625	---	---	0.35	1.96	---	3.39	2.57
Heptane, 2,4-dimethyl-	5.082	0.55	---	---	---	---	---	---
Methylpyrazine	5.158	---	---	---	---	0.96	1.07	0.74
Furfural	5.401	---	---	---	1.14	2.58	2.69	2.39
2,4-Dimethyl-1-heptene	5.538	0.26	---	---	---	---	---	---
2-Methylene-hexanal	6.087	---	---	---	1.01	---	1.36	1.13
p-Xylene	6.238	1.06	1.97	0.49	---	---	---	---
Styrene	6.787	2.92	---	---	---	---	---	---
o-Xylene	6.847	---	0.76	0.18	---	---	---	---
Heptanal	7.076	---	---	---	0.26	0.55	---	0.37

Acetylfuran	7.35	---	---	---	---	---	0.36	0.34
γ-Terpinen	7.639	---	---	---	0.18	---	0.36	0.3
Benzene, (1-methylethyl)-	7.715	0.9	---	---	---	---	---	---
α-Thujene	7.776	---	---	---	0.55	1.13	0.77	---
α-Phellandrene	7.791	---	---	---	---	---	---	0.82
α-Pinene	7.974	---	0.38	0.11	13.02	8.55	11.38	13.24
Camphene	8.385	---	---	---	0.59	0.75	0.83	0.96
Benzene, propyl-	8.537	0.32	---	---	---	---	---	---
Dehydrosabinene	8.552	---	---	---	0.95	1.8	1.82	2.04
Cyclohexane, 1,1,2,3-tetramethyl-	8.568	---	---	0.21	---	---	---	---
Benzaldehyde	8.735	1.52	---	---	3.9	5.95	3.77	4.03
Benzene, 1-ethyl-3-methyl-	8.765	---	0.67	0.54	---	---	---	---
5-methyl-2-furfural	8.842	---	---	---	---	0.54	0.58	0.49
Mesitylene	8.963	0.5	1.19	0.4	---	---	---	---
p-Cymene	9.024	---	---	---	0.76	1.18	0.51	0.64
Sabinene	9.116	---	---	---	0.46	0.95	0.51	0.54
β-Pinene	9.207	---	---	---	3.29	0.94	0.55	4.56
Benzene, 1-ethyl-2-methyl-	9.298	---	---	0.32	---	---	---	---
α-Methylstyrene	9.359	4.13	0.43	---	---	---	---	---
2-Pentylfuran	9.648	---	---	---	1.49	2.46	1.69	1.58
Benzene, 1,2,3-trimethyl-	9.694	2.31	---	1.9	---	---	---	---
Octanal	9.892	0.18	---	0.35	---	---	---	---
1-Methylformylpyrrole	10.029	---	---	---	---	1.73	1.82	1.57
3-Carene	10.196	---	---	---	2.33	2.21	1.57	2.06
Pinonene	10.379	---	---	---	0.22	---	0.38	0.39
Benzene, 1,2,4-trimethyl-	10.547	---	---	1.67	---	---	---	---
o-Cymene	10.622	---	---	---	0.44	1.44	1.49	1.41
Limonene	10.744	---	---	---	0.72	1.08	0.97	0.85
Indane	10.927	---	---	0.43	---	---	---	---
Cyclohexane, 1,1-dimethyl-2-propyl-	11.14	---	0.42	0.4	---	---	---	---
Salicylaldehyde	11.155	---	---	---	0.14	---	---	---
Benzene, 1-methyl-3-propyl-	11.414	---	0.98	0.86	---	---	---	---
Naphthalene, decahydro-	11.49	---	---	0.43	---	---	---	---
2,3,6-Trimethyltetradecane	11.612	7.81	3.7	---	---	---	---	---
Benzene, 2-ethyl-1,4-dimethyl-	11.627	---	---	0.84	---	---	0.93	---
Decane, 3,6-dimethyl-	11.779	1.9	---	---	---	---	---	---
1-Pentacontanol	11.795	---	---	0.41	---	---	---	---
1-Propanone, 1-phenyl-	11.855	0.49	---	---	---	---	---	---
Benzene, 1-methyl-4-propyl-	11.871	---	---	0.39	---	---	---	---
Decane, 3-methyl-	11.993	---	---	0.18	---	---	---	---
Benzene, 1-ethyl-3,5-dimethyl-	12.175	---	0.6	1.13	---	---	---	---
Benzene, 1-ethyl-2,3-dimethyl-	12.251	2.14	---	1.44	---	---	---	---
m-Cymenene	12.343	---	0.77	---	0.43	0.61	0.48	0.49
Cyclopentene,3,5-dimethylene-1,4,4-trimethyl-	12.419	---	1.45	---	---	---	---	---
p-Cymenene	12.54	---	---	---	---	1.31	1.48	1.35
4,7-Methano-1H-indene, octahydro-	12.632	2.19	0.9	0.69	---	---	---	---
Decane, 3-methyl-	12.86	---	---	1.57	---	---	---	---
Decane	12.997	---	---	---	---	---	0.6	---
Decane, 3,7-dimethyl-	13.104	---	1.37	---	---	---	---	---
trans-Decalin, 2-methyl-	13.134	---	1.26	0.53	---	---	---	---
Fenchol	13.256	---	---	---	0.24	---	---	0.28
Benzene, 1-ethyl-2,3-dimethyl-	13.317	---	---	1.63	---	---	---	---
Benzene, 1,2,4,5-tetramethyl-	13.454	0.56	1.35	1.56	---	---	---	---
Benzyl isocyanate	13.53	0.21	---	---	---	---	---	---
Naphthalene, decahydro-2-methyl-	13.621	---	---	1.35	---	---	---	---
1-Methyl-2-phenylcyclopropane	13.987	---	---	0.65	---	---	---	---
Benzene, 1,3-diethyl-5-methyl-	14.154	---	---	0.47	---	---	---	---
L-camphor	14.185	---	---	---	0.5	0.77	0.58	---
Benzene, 1-ethenyl-4-ethyl-	14.306	0.18	---	1.04	---	---	---	---
Benzene, 1-ethyl-3,5-dimethyl-	14.382	0.2	1.18	1.32	---	---	---	---
Benzene, 1,3-bis(1-methylethyl)-	14.443	0.27	---	---	---	---	---	---

Pinocarvone	14.717	---	---	---	---	0.3	0.3	0.41
Naphthalene, decahydro-2,6-dimethyl-	15.159	---	---	0.71	---	---	---	---
Naphthalene	15.28	0.19	0.65	0.63	---	---	---	---
Alpha terpineol	15.524	---	---	---	2.3	2.88	1.94	1.9
Benzene, (2-methyl-1-butenyl)-	15.57	---	---	0.31	---	---	---	---
Myrtenal	15.692	---	---	---	0.64	0.82	---	---
Tridecane, 6-methyl-	15.767	1.08	2.78	---	---	---	---	---
Naphthalene, decahydro-2,6-dimethyl-	15.904	---	0.49	---	---	---	---	---
Decanal	15.935	0.31	---	---	---	---	---	---
Levoverbenone	15.935	---	---	---	0.99	1.79	1.57	1.71
Undecane, 2,6-dimethyl-	16.163	0.32	---	1.29	---	---	---	---
Cyclohexane, 2-butyl-1,1,3-trimethyl-	16.376	---	0.73	---	---	---	---	---
Methenamine	16.392	---	---	1.13	9.27	19.34	17.56	16.38
Dodecane, 4,6-dimethyl-	16.787	0.49	---	---	---	---	---	---
Cuminaldehyde	16.909	---	---	---	---	---	0.27	---
Benzene, (1-methyl-1-butenyl)-	17.153	---	---	0.74	---	---	---	---
Naphthalene, 1,2,3,4-tetrahydro-6-methyl-	17.548	---	---	1.55	---	---	---	---
Nonane, 3-methyl-	17.792	---	---	1.05	---	---	---	---
Tridecanol, 2-ethyl-2-methyl-	17.837	0.99	---	---	---	---	---	---
1-Iodotridecane	18.005	8.22	5.64	---	---	---	0.32	---
Undecane	18.385	0.87	---	---	---	---	---	---
Tridecane, 6-methyl-	18.538	---	1.04	1.98	---	---	---	---
Naphthalene, 1-methyl-	18.827	---	---	0.98	---	---	---	---
Naphthalene, 1,2,3,4-tetrahydro-1,4-dimethyl-	19.025	---	---	1.32	---	---	---	---
10-Methylnonadecane	19.07	---	0.66	---	---	---	---	---
Tridecane	19.238	2.89	---	---	---	---	---	---
Tetradecane, 1-iodo-	19.253	1.03	---	---	---	---	---	---
Dodecane, 1-iodo-	19.497	0.99	---	---	---	---	---	---
Nonyl tetradecyl ether	19.74	---	0.42	---	---	---	---	---
2,6,10-Trimethyltridecane	19.953	0.25	---	---	---	---	---	---
1H-Inden-1-one, 2,3-dihydro-3,3-dimethyl-	20.379	2.03	---	---	---	---	---	---
Benzene, 1,3-bis(1-methylethenyl)-	20.547	0.25	---	---	---	---	---	---
Dodecane, 2,7,10-trimethyl-	20.562	---	---	1.39	---	---	---	---
1H-Indene, 2,3-dihydro-1,1,3-trimethyl-	20.989	---	---	1.17	---	---	---	---
Methyleugenol	21.308	---	---	---	0.18	0.15	---	---
Longifolene	21.415	---	---	---	0.34	0.64	0.72	0.38
1,3-DIACETYLBENZENE	22.023	2.8	---	---	---	---	---	---
2,6,10-Trimethyltridecane	22.724	---	0.41	1.37	---	---	---	---
Hexadecane, 1-iodo-	22.754	0.32	---	---	---	---	---	---
Benzoquinone, 2,6-di-(1,1-dimethylethyl)	22.906	---	1.55	---	---	---	---	---
α-Cedrene	23.181	---	---	---	---	---	---	0.19
3-Ethyl-2,6,10-trimethylundecane	23.195	0.49	---	---	---	---	---	---
Undecane, 3,8-dimethyl-	23.454	0.57	0.45	---	---	---	---	---
5,5,7,7-Tetraethylundecane	23.576	---	3.74	0.42	---	---	---	---
α-Muurolene	23.744	---	---	---	---	0.18	---	---
Pentadecane	23.789	0.49	0.51	---	---	---	---	---
Butylated Hydroxytoulene	24.033	---	8.33	1.01	---	---	0.72	---
δ-Cadinene	24.307	---	---	---	0.15	0.23	---	---
Hexadecane	24.535	0.21	---	---	---	---	---	---
Heptadecane	24.642	---	---	---	---	---	0.26	---
Octadecane, 3-ethyl-5-(2-ethylbutyl)-	24.642	---	1.5	---	---	---	---	---
Cyclohexane, (1,3-dimethylbutyl)-	24.885	---	---	0.63	---	---	---	---
Ethanol, 2-(dodecyloxy)-	25.159	---	---	0.39	---	---	---	---
Nonadecane	26.012	1.88	0.94	3.54	---	0.24	1.09	1.63
Tetracontane, 3,5,24-trimethyl-	27.427	---	---	0.33	---	---	---	---
Crocetane	28.371	---	0.38	1.03	---	---	---	0.43
Methoxyacetic acid, 2-tridecyl ester	28.386	---	---	---	---	---	0.48	---
Carbonic acid, tetradecyl vinyl ester	28.493	---	---	0.13	---	0.8	---	---

Heptadecane, 4-methyl-	29.421	---	0.73	---	---	0.56	---	---
1-Pentadecanethiol	29.497	---	---	0.23	---	---	---	---
Eicosane	30.365	---	0.58	2.57	---	0.37	1.42	2.03
Phytane	30.563	---	0.39	---	---	---	0.39	0.46
Heptadecane, 2,6,10,15-tetramethyl-	30.578	---	---	0.73	---	---	---	---
Tridecane, 4-cyclohexyl-	31.674	---	---	0.2	---	---	---	---
1-(2-Propen-1-yloxy)dodecane	31.826	---	---	0.1	---	---	---	---
Heneisocane	32.405	---	---	---	0.36	0.4	1.36	1.58
7,9-Di-tert-butyl-1-oxaspiro(4,5)deca-6,9-diene-2,8-dione	32.846	---	0.35	---	---	---	---	---
Dotriacontane, 1-iodo-	32.907	---	0.41	---	---	0.45	---	---
Hexadecanoic acid, methyl ester	32.922	---	---	0.41	---	---	---	0.19
Cembrene	33.166	---	---	---	0.14	---	---	---
Tetracosane	35.525	---	---	---	0.21	0.52	1.72	---
Heptadecane, 2,6,10,15-tetramethyl-	36.195	---	---	0.97	---	0.61	1.3	0.99
9-Methylnonadecane	38.037	---	0.34	0.47	---	1.49	---	1.47
Pentacosane	39.589	---	---	0.18	0.99	1.03	0.92	0.91
Heptacosane	40.701	0.22	---	0.12	---	1.65	1.7	1.33
